# Host-pathogen interactions involved in erythrocyte invasion by *Francisella tularensis*

**DOI:** 10.3389/fcimb.2025.1664733

**Published:** 2025-09-23

**Authors:** Rori M. Schreiber, Luke D’Cunha, Mackenzie Hall, Anthony DeBastiani, Deanna M. Schmitt, Stuart Cantlay, Joseph Horzempa

**Affiliations:** ^1^ Department of Biological Sciences, West Liberty University, West Liberty, WV, United States; ^2^ West Virginia University Shared Research Facilities, West Virginia University, Morgantown, WV, United States

**Keywords:** *Francisella tularensis*, GcvT, spectrin, Band 3, glycophorin A, PdpC, Type VI Secretion System, Ankyrin-1

## Abstract

*Francisella tularensis* is a gram negative, facultative intracellular bacterium that causes the zoonotic disease tularemia. *F. tularensis* is capable of invading mammalian erythrocytes, a phenomenon that enhances colonization of ticks following a blood meal. The colonization of these blood-sucking arthropods presumably enhances transmission to mammalian hosts and increases the persistence of this bacterium within the environment. Therefore, we sought to elucidate host-pathogen interactions involved in erythrocyte invasion by *F. tularensis*. In this study, we identified the red blood cell (RBC) membrane protein, Band 3, is required for erythrocyte invasion by *F. tularensis.* Erythrocyte proteins that complex with Band 3 were also evaluated for their role in erythrocyte invasion. While Glycophorin A impedes invasion, Ankyrin-1, the peripheral membrane protein that links Band 3 to the spectrin cytoskeleton, is required for erythrocyte invasion. Furthermore, expression of the recombinant cytoplasmic domain of Band 3 in *F. tularensis* LVS was used to identify interacting bacterial proteins. Here, we identified that the *F. tularensis* Glycine Cleavage Protein T, GcvT, interacts with the cytoplasmic domain of Band 3. A mutational analysis confirmed GcvT is required for RBC invasion by *F. tularensis* validating the finding that GcvT interacts with Band 3. Lastly, we determined that the *F. tularensis* Type VI Secretion System Effector Protein, PdpC, interacts with the erythrocyte cytoskeletal protein, Spectrin, alpha chain, likely contributing to the ability of *F. tularensis* to invade erythrocytes.

## 1 Introduction

*Francisella tularensis* is a gram negative, non-motile bacterium that causes the zoonotic disease tularemia (Oyston, 2008; Degabriel et al., 2023). *F. tularensis* is considered a Category A bioterrorism agent by the Centers for Disease Control and Prevention (CDC) as this bacterium can be aerosolized and causes a potentially fatal infection with as little as ten colony forming units (CFU) (CDC, 2018). The natural transmission of this bacterium is commonly mediated via blood-sucking arthropod vectors such as ticks, mosquitoes, and biting flies that can transmit this infectious agent to small rodents, lagomorphs, and humans (Akimana and Kwaik, 2011). During mammalian infection, *F. tularensis* replicates within host cells such as macrophages, neutrophils, hepatocytes, and alveolar lung epithelial cells which is important for the pathogenesis of this bacterium (Celli and Zahrt, 2013).

In addition to surviving and replicating within host phagocytes, *F. tularensis* is also capable of invading mammalian erythrocytes (Horzempa et al., 2011). Erythrocyte invasion by *F. tularensis* has been shown to enhance colonization in ticks following a blood meal (Schmitt et al., 2017). While entry into both phagocytes and non-phagocytic nucleated mammalian cells requires the host endocytic degradative pathway, erythrocytes completely lack this machinery (Schekman and Singer, 1976; Clemens et al., 2005). Unlike the majority of host cells that produce a cytoskeleton composed of dynamic actin filaments and microtubules, erythrocytes possess a flexible spectrin network tethered by small f-actin bundles that are linked to the erythrocyte membrane (Nigra et al., 2020). The linkage of the cytoskeleton to the erythrocyte membrane can be attributed to two major integral protein complexes: the Band 3/Ankyrin complex and the 4.1R complex (Salomao et al., 2008; Mankelow et al., 2012). The Band 3/Ankyrin complex is composed of integral membrane proteins such as Band 3 and Glycophorin A (GPA) that associate with the spectrin cytoskeleton via Ankyrin-1 (Mankelow et al., 2012). The 4.1R complex consists of the membrane protein Band 3 linked to the cytoskeleton via actin bundles and protein 4.1R (Salomao et al., 2008; Mankelow et al., 2012).

Few intracellular pathogens are capable of invading mammalian erythrocytes, and most require proteins involved in the Band 3/Ankyrin complex or 4.1R complex to facilitate invasion (Horzempa et al., 2011; Deng et al., 2012; Alam et al., 2016; Lu et al., 2022). *Plasmodium falciparum* secretes over 400 proteins into erythrocytes, including erythrocyte binding-link (EBL) and reticulocyte binding-like proteins (RBL) that interact with Spectrin, Actin, Ankyrin-1, Band 3, Glycophorin A (GPA) and protein 4.1R to facilitate red blood cell invasion (Mohandas and An, 2012; Lu et al., 2022). The *Bartonella* sp. Type IV Secretion System proteins, TrwJ1 and TrwJ2, interact with Band 3 to adhere to red blood cells, allowing for subsequent invasion (Deng et al., 2012). Previous research also suggests an interaction between *Bartonella* sp. and Glycophorin A and B could be used as a mechanism for erythrocyte invasion by this bacterium (Deng et al., 2012).

Because of the distinct cytoskeletal architecture of erythrocytes, and since cytoskeletal fibers are often implicated in events involving extracellular uptake, Spectrin and Band 3 may therefore be required for invasion of erythrocytes by *F. tularensis*. Previous studies have shown that an animal toxin (venom) from *Pseudechis guttatus* inhibits erythrocyte invasion by *F. tularensis* (Schmitt et al., 2017). This venom specifically binds the erythrocyte surface protein, Band 3, which leads to spectrin impairment (Yau et al., 2012). Therefore, the preclusion of invasion during venom treatment further implicates Band 3. However, inhibitors of actin polymerization and depolymerization did not affect erythrocyte invasion by *F. tularensis in vitro* (Schmitt et al., 2017) implicating the Band 3/Ankyrin complex, but not the 4.1R complex.

*F. tularensis* utilizes a Type VI Secretion System (T6SS) apparatus during infection to deliver effector proteins into host cells that promote intracellular survival and replication (Bröms et al., 2010; Spidlova and Stulik, 2017). The *F. tularensis* T6SS and effector proteins are encoded by the Francisella Pathogenicity Island, a 30-kb duplicate genetic locus that is required for pathogenesis of this bacterium (Bröms et al., 2010). The *F. tularensis* T6SS and effectors mediate phagosomal escape and intracellular replication within macrophages, as well as activation of host immune responses like AIM2 inflammasomes (Bröms et al., 2010; Brodmann et al., 2017, 2021). The T6SS is also required by *F. tularensis* for erythrocyte invasion (Schmitt et al., 2017). Invasion of mammalian erythrocytes by *F. tularensis* is facilitated by the T6SS effector protein, PdpC (Schmitt et al., 2017; Cantlay et al., 2022). PdpC also plays a pivotal role in the pathogenesis of *F. tularensis*, being necessary for escaping the phagosome of macrophages, intracellular replication, and disease in mouse models (Lindgren et al., 2013; Clemens et al., 2018; Brodmann et al., 2021).

In this study, we sought to investigate interactions between *F. tularensis* and mammalian erythrocytes that contribute to erythrocyte invasion. Utilizing a gentamicin protection assay, we identified the erythrocyte glycoprotein, Band 3, is required for *F. tularensis* erythrocyte invasion. Further investigation identified the erythrocyte protein Glycophorin A may act to inhibit invasion, while the erythrocyte protein, Ankyrin-1, is required for bacterial invasion. We have identified bacterial proteins that potentially interact with the cytoplasmic domain and membrane associated domains of Band 3 *in vitro.* Of the identified proteins, the Glycine Cleavage Protein T, GcvT, was found to interact with the cytoplasmic domain of Band 3 and suggested to contribute to erythrocyte invasion. Lastly, we identified the *F. tularensis* T6SS Effector Protein, PdpC that is required for invasion, interacts with the erythrocyte protein, Spectrin, alpha chain. These findings highlight a key role for the involvement of the erythrocyte Band 3/Ankyrin/Spectrin complex in *F. tularensis* erythrocyte invasion and a direct link between this complex and an effector of the T6SS.

## 2 Materials and methods

### 2.1 Bacterial strains and growth conditions

Bacterial strains used in this study are listed in **Table 1**. F*. tularensis* LVS was grown on chocolate II agar plates and incubated at 37°C with 5% CO_2_ for 2–3 days. Bacteria from the agar plates were then used to inoculate either Tryptic Soy Broth supplemented with 0.1% L-cysteine (TSBc) or Chamberlain’s Chemically Defined Medium (CDM) (Chamberlain, 1965). Cultures were incubated at 37°C with agitation and grown to stationary phase. When indicated, media were supplemented with 3-4% potassium chloride (KCl). *Escherichia coli* was grown on Luria Bertani (LB) agar plates and incubated at 37°C for 1 day. Broth cultures were grown to stationary phase by inoculating LB broth with *E. coli* bacteria and incubating overnight at 37°C with agitation. For bacterial strains harboring plasmids, the media was supplemented with kanamycin at 10 μg/ml for *F. tularensis* LVS or ampicillin at 100 μg/ml for *E. coli.*

**Table 1:** Bacterial strains, plasmids, and sequence primers used in this study.

Strain, plasmid, or primer	Description or sequence	Source or reference
*F. tularensis* Strains		
LVS	*F. tularensis* subsp. *holarctica* live vaccine strain	Karen Elkins
LVS/pCDB3	LVS containing the pCDB3 vector	This study
LVS/pCDB3-emGFP	LVS containing the pCDB3-emGFP vector	This study
LVSΔ*gvct*	LVS *gcvT* deletion mutant	(Brown et al., 2014)
LVSΔ*gvct*/pMB1:*gcvT*	LVS *gcvT* deletion mutant containing the *gcvT* complementation vector	(Brown et al., 2014)
LVS *pdpC*-null	LVS *pdpC* deletion mutant	(Cantlay et al., 2022)
LVS/pKHEG	LVS containing the pKHEG vector	(Haggerty et al., 2024)
*E. coli* Strains		
*E. coli* DH5α	*fhuA2 Δ(argF-lacZ)U169 phoA glnV44 ϕ80Δ(lacZ)M15 gyrA96* *recA1 relA1 endA1 thi-1 hsdR17*	Takara Bio USA
DH5α/pKHEG	*E. coli* NEB-5-α strain containing pKHEG vector	(Haggerty et al., 2024)
Plasmids		
pKHEG	Plasmid containing emGFP under control of FGRp with 580N	(Haggerty et al., 2024)
pCDB3	Plasmid containing the cytoplasmic domain of Band 3 under the control of FGRp	This study
pCDB3-emGFP	Plasmid containing the cytoplasmic domain of Band 3 linked to emGFP under the control of FGRp	This study
pGEM T-Easy	Pre-linearized vector system under the control of the lac promoter (Promega)	Promega
Primers		
cdb3F	5’-CATGCATATGGGTGGATCAGGTGGATCAATGGAGGAGCTGCAGGACGA-3’	IDT^1^ This study
cdb3R	5’-CATGCATATGGGTGGATCAGGTGGATCACTTATAGAAAGAGGAATCTGGCTTGGC-3’	IDT This study
cdb3-gfpF	5’-CATGCATATGGGTGGATCAGGTGGATCAATGGAGGAGCTGCAGGACGA-3’	IDT This study
cdb3-gfpR	5’-CATGCATATGTGATCCACCTGATCCACCCTTATAGAAAGAGGAATCTGGCTTGGC-3’	IDT This study
AnkF	5’-TTCTGCTTCCCTGTCTCCAT -3’	Jackson Laboratories
AnkR	5’-AGAGAGTTGGCTGGGTGGTA-3’	Jackson Laboratories

### 2.2 Human erythrocyte purification

Human erythrocytes were isolated from whole blood obtained from donors by West Liberty University’s Medical Lab Science Department. PBS was added to an equal volume of the blood sample and underlaid with Ficoll to separate erythrocytes from white blood cells using density gradient centrifugation. The plasma was removed, and erythrocytes were washed in McCoy’s 5A medium supplemented with 10% human AB serum and 25 mM HEPES buffer (red blood cell media). Erythrocytes were counted using a hemocytometer and resuspended in red blood cell media to achieve the desired concentration (Horzempa et al., 2011).

### 2.3 Fab2 fragment generation

Fab2 and Fc fragments were obtained from unconjugated rabbit IgG polyclonal antibody reactive against human Band 3 (Invitrogen, REF: PA5-80030; Novus, REF: NBP1-70433) and human Glycophorin A (CD235a, Thermo Scientific CAT # 85882). The antibodies were subject to cysteine protease digestion (Genovis FragIT, Pr. No: A2-Fr2-005; Genovis FabRICATOR Immobilized CAT # A0-FR6-010) following the manufacturers protocol to generate Fc fragments and Fab2 fragments. To isolate Fab2 fragments, Fc fragments were captured using IgG-Fc magnetic affinity beads and the Fab2 fragments were eluted (CaptureSelect IgG-Fc Magnetic Agarose Beads, Thermo Scientific). The immobilized Fc fragments produced from antibody digestion were also isolated according to the instructions of the manufacturer and were used as controls. Fab2 and Fc fragment concentrations were quantified utilizing the QuickStart Bradford Protein Assay following the manufacturers protocol. Isolated Fab2 and Fc fragments were stored at -20°C until further analysis could be conducted.

### 2.5 Animals and ethics approval

All studies using mice were approved by the West Liberty University Animal Care and Use committee and were conducted using ethical standards (OLAW assurance number D21-01113). WB/Re (WB*) +/+* and *nb/+* inbred mice (JAX stock #000453), 6–8 weeks old, were purchased from The Jackson Laboratory (Bar Harbor, ME). These mice contain a spontaneous mutation in the Ankyrin-1 gene (Ank1) that results in the introduction of a premature stop codon and subsequent truncation of the Ankyrin-1 protein found in erythrocytes (Birkenmeier et al., 2003). The *nb* mutation was maintained in the heterozygous state in the WB/Re strain. Homozygotes (WB/Re *nb/nb*) for this study were obtained by crossing WB/Re heterozygotes and were identified by their light orange color and small size compared to wild type and heterozygotes (The Jackson Laboratory). Confirmation of ankyrin deficiency in murine erythrocytes was obtained via western blotting (rabbit anti-Ankyrin 1, Invitrogen CAT # PA5-63372, 1:1000) and Sanger sequencing of the amplicons generated using the AnkF and AnkR primers (data not shown). Controls consisted of normal WB/Re *+/+* mice. In this study, 6 WT mice and 3 *nb/nb* mice were used. Murine blood was obtained by neonatal decapitation with veterinary-grade isoflurane euthanasia from neonatal mice 2–3 days old (Seman et al., 2020). Approximately 150–200 microliters of blood could be obtained from neonates. EDTA (0.5 mM) was used as an anticoagulant. Murine erythrocytes were then purified as previously described (Horzempa et al., 2011).

### 2.6 Gentamicin protection assays

Erythrocytes isolated from blood samples (as described above) were diluted to a concentration of 1 x 10^7^ cells/ml in red blood cell media and aliquoted into a 96-well V-bottom dish. For experiments involving purified recombinant Band 3 (rBand 3), the bacteria and rBand 3 (10 μg/ml) were incubated for 1 hour at 37 °C and 5% CO_2_, after which the bacteria/rBand 3 mixture and erythrocytes were combined and incubated. For assays using Glycophorin A (GPA) and Band 3 Fab2 fragments, erythrocytes were treated with GPA Fab2 fragments (0.2 mg/ml) or Band 3 Fab fragments (2 mg/ml) and incubated for 1 hour at 37°C with 5% CO_2._ CDM was inoculated with either *F. tularensis* LVS, *F. tularensis* LVSΔ*gcvT*, or *F. tularensis* LVSΔ*gcvT/*pMB1:*gcvT* and incubated at 37°C with agitation for at least 16 hours prior to the start of an experiment. The CDM growth medium was used as it promotes erythrocyte invasion while also producing minimal background levels of viable extracellular bacteria after gentamicin treatment (Haggerty, 2025). Bacteria were diluted to a targeted multiplicity of infection (MOI) of 12.5 (1.25 x 10^8^ CFU/mL) in red blood cell media (Schmitt et al., 2017) and were incubated at 37°C with 5% CO_2_ for at least 15 minutes. The actual MOI of the experiment was determined by serial diluting and plating for CFU. Bacteria and erythrocytes were incubated for 2–3 hours at 37°C with 5% CO_2._ After incubation, erythrocytes were pelleted and washed with PBS. Gentamicin (25 μg/ml in PBS) was pre-warmed and added to all experimental wells. The plate was incubated at 37°C with 5% CO_2_ for 45 minutes to 1 hour. Following incubation, the plate was centrifuged at 100 x g for 5 minutes, and the supernatant was discarded. Wells were washed with PBS and erythrocytes were lysed with 0.02% sodium dodecyl sulfate (SDS). Lysates were plated onto chocolate II agar and incubated at 37°C with 5% CO_2_ for 3–5 days (Horzempa et al., 2011; Schmitt et al., 2017) to determine CFU. Wells lacking erythrocytes (-RBC) were used to determine the efficacy of the gentamicin in killing *F. tularensis* LVS which was negligible for all experiments. The average CFU from -RBC wells was considered to be background and this value was subtracted from the experimental groups in each experiment.

### 2.7 Construction of pCDB3 plasmid

Primers and plasmids used in this study are listed in **Table 1**. Coding sequence for the cytoplasmic domain of the human Band 3 gene, *SLC4A1*, was amplified using cDNA from the donor plasmid pDONR221_SLC4A1 (RESOLUTE Consortium & Giulio Superti-Furga; Addgene plasmid # 132180, RRID: Addgene_132180) using primer sets cdB3F and cdB3R. PCR products were excised from the gel and subject to purification using the Monarch DNA Gel Extraction Kit (New England Biolabs). The purified PCR product and plasmid pKHEG were digested with NdeI. The plasmid vector pKHEG was simultaneously incubated with rSAP to prevent self-ligation and then heat inactivated at 65°C for 10 minutes. The digested plasmid vector and insert were ligated using T4 ligase at 15°C for 16 hours and subsequently heat-inactivated at 65°C for 10 minutes. Competent NEB 5α *E. coli* cells were transformed with the pCDB3 plasmid according to the protocol of the manufacturer. Plasmids were isolated from cultures utilizing the Monarch Plasmid Mini Prep Kit (New England Biolabs) and screened via restriction digest using the enzyme NdeI to confirm the plasmids possessed the desired insert. A subsequent restriction digest using the enzyme KpnI was performed to ensure the insert was in the same direction as the FGRp promotor (Horzempa et al., 2008). Plasmids possessing the desired product were then electroporated into *F. tularensis* LVS as previously described (Horzempa et al., 2011).

### 2.8 Construction of pCDB3-emGFP plasmid

The generation of the pCDB3-emGFP plasmid was performed using a similar approach to the construction of pCDB3 with the following exceptions. Here, the same fragment of *SLC4A1* was amplified from pDONR221_SLC4A1 using the primer set cdb3-gfpF and cdb3-gfpR leading to the addition of coding sequence for a 3’ linker peptide. The amplicon produced was excised from the gel and purified using the Monarch DNA Gel Extraction kit (New England Biolabs). The purified amplicon and pKHEG were digested using NdeI and the plasmid was also incubated with rSAP. Reactions were subsequently heat-inactivated at 65 °C for 10 minutes. The plasmid and amplicon restriction fragments were ligated using T4 ligase and then used to transform competent *E. coli* NEB 5α. Plasmids confirmed to contain the desired product in the appropriate orientation were then electroporated into *F. tularensis* LVS as previously described (Horzempa et al., 2008).

### 2.9 Western blots

Western blotting was carried out similarly as previously described (Haggerty et al., 2024). Briefly, bacterial cultures were grown to stationary phase and normalized to the same OD_600_ value to account for differences in bacterial growth. Bacterial lysates were generated by adding an equal volume of 2x Laemmli buffer supplemented with 5% 2-mercaptoethanol and boiling at 95°C for 5 minutes. Samples were electrophoresed in a 4-15% Mini-PROTEAN TGX gel (BioRad Laboratories) and were subsequently electroblotted onto a nitrocellulose membrane. This membrane was blocked with PBS containing 0.5% casein, 0.5% bovine serum albumin, 100 mg/L phenol red, and 0.02% sodium azide pH 7.4 for 30 minutes. The nitrocellulose membrane was then incubated with the primary antibody (rabbit anti-Band 3, Invitrogen CAT # PA5-80030, 1:1000; rabbit anti-emGFP, Invitrogen CAT # A-11122, 1:1000, rabbit anti-Ankyrin 1, Invitrogen CAT # PA5-63372, 1:1000) overnight and shaking at room temperature. The membrane was then washed 3 times with PBS and subsequently incubated with the secondary antibody (goat anti-rabbit alkaline phosphatase, Invitrogen CAT # 31340, 1:1000) for 1 hour shaking at room temperature. The membrane was washed 2 times with PBS and once with 0.05 M Tris buffer, pH 8.0 and then developed by incubating with Fast Red and Naphthol AS-MX phosphate disodium salt dissolved in Tris buffer until red protein bands appeared (Haggerty et al., 2024).

### 2.10 Fluorescence microscopy

Specimens were imaged using an Olympus IX73 microscope with an ORCA-Flash4.0 LT+ Digital CC11440-42U CMOS camera (Hamamatsu) and a 100x NA 1.45 phase objective. *F. tularensis* LVS strains expressing Band 3-emGFP were inoculated from chocolate II agar plates into TSBc or CDM supplemented with kanamycin at 10 μg/ml at 37°C for 12–16 hours with agitation. Bacterial cultures were added to 1% agarose pads and upon which a cover slip was placed. Similar exposure times were used for all images, and brightness and contrast was adjusted uniformly across all images.

### 2.11 Co-immunoprecipitation assays

Freeze dried Dynabeads (M-270 Epoxy; Invitrogen) were equilibrated by resuspending 5 mg of these beads (~3.3 x 10^8^ beads/ml) in Buffer A (0.1 M sodium phosphate, pH 7.4) and incubating for 10 minutes at room temperature with rotation. Beads were placed on a magnet and the supernatant was discarded. The beads were resuspended in Buffer A one more time before being coupled to the antibody. Equilibrated beads were resuspended in an equal volume of Buffer A and ligand (rabbit anti-Band 3 antibody, 100 μg, Invitrogen CAT # PA5–80030 or rabbit anti-PdpC antibody, 100 μg, BEI resources, NR-4379) and vortexed. The same volume of Buffer B (3.0 M ammonium sulfate) was then added, followed by end-over-end rotation for 16–24 hours at 37°C. Beads were separated by a magnet and the supernatant was removed. Beads were washed 4 times with Buffer E1 (PBS) and then resuspended in E1 to achieve the desired concentration (~2.9 x 10^8^ beads/ml). Overnight cultures of *F. tularensis* LVS, LVS/pCDB3, LVS *pdpC*-null were normalized to an OD_600_ of 3.0 and sonicated 3 times by 15 seconds to generate lysates. Lysates were added to the antibody conjugated beads and incubated with end-over-end rotation for 2 hours at 4°C. Beads were placed on a magnet and the supernatant was removed. Beads were washed 3 times with Buffer E1 and resuspended in 2x Laemmli buffer with 5% 2-mercaptoethanol. Beads were boiled at 95°C for 5 minutes and placed on a magnet to separate the beads. The supernatant was collected and stored at -20°C.

### 2.12 GFP-affinity pull down assays

GFP-Trap Magnetic agarose beads (Chromotek) were equilibrated by resuspending beads in ice-cold dilution buffer (Chromotek) 3 times. Overnight stationary phase broth cultures of *F. tularensis* strains containing pCDB3-emGFP or pKHEG were normalized to the lowest OD_600_ and sonicated to generate whole cell lysates. Lysates were added to equilibrated beads and incubated for 1 hour at 4°C with end-over-end rotation. The beads were separated with a magnet and the supernatant was removed. Beads were washed 3 times with Wash buffer (Chromotek) and transferred to a new tube after the final wash. The supernatant was removed, and beads were resuspended in 80μl of 2x Laemmli buffer supplemented with 5% 2-mercaptoethanol. To dissociate complexes from the beads, beads were boiled for 5 minutes at 95°C. The beads were separated with a magnet and the supernatant was stored at -20°C.

### 2.13 In-gel proteomic digestion

Digestion reagents were prepared as follows: Trypsin Protease stock solution was prepared by reconstituting 20 µg of the lyophilized material in 20 µL of 0.1% acetic acid. Aliquots of the trypsin stock solution were transferred to individual microcentrifuge tubes and stored at -80°C until needed. Destaining solution for Coomassie blue was prepared by dissolving 80 mg of ammonium bicarbonate in 40 mL of a 50:50 solution of acetonitrile and ultrapure water. Destaining solution for silver-stained gel was prepared by mixing a solution of 50% ultrapure water, 10% acetic acid, and 40% methanol. Digestion buffer was prepared by dissolving 10 mg of ammonium bicarbonate in 5 mL of ultrapure water. Reducing buffer was prepared immediately prior to use by mixing 3.3 µL of TCEP with 30 µL of digestion buffer for each digestion sample. Alkylation buffer was prepared immediately before use while avoiding exposure to light by preparing a 500 mM solution of IAA in ultrapure water with dilution of this stock solution by mixing 7 µL of the stock solution with 28 µL of digestion buffer for each digestion sample. Activated trypsin was prepared immediately prior to use by diluting 5 µL of trypsin stock solution with 45 µL of digestion buffer for each digestion sample.

Protein bands of interest were excised from the gel and stored at -20°C until use. Bands were destained in destaining solution for 30 minutes at 37°C with agitation. The destaining solution was removed and repeated until the stain was removed from the samples. Reducing buffer was added to samples and incubated at 60°C for 10 minutes. Reducing buffer was removed and alkylation buffer was added to samples and incubated in the dark at room temperature for 60 minutes. Following incubation, alkylation buffer was removed, and samples were washed twice with destaining buffer at 37°C for 15 minutes with agitation. Samples were digested by adding acetonitrile and incubating samples for 15 minutes at room temperature. Acetonitrile was removed and samples were allowed to dry for 10 minutes at room temperature. Once samples were dried, activated trypsin and digestion buffer were added to samples and incubated at 30°C overnight. After incubation, activated trypsin and digestion buffer were removed and 1% formic acid solution was incubated with the samples for 5 minutes. Once the formic acid solution was removed, samples were subject to purification using Pierce C18 spin columns (Thermo Fisher Scientific). Samples were stored at -20°C until further analysis could be conducted.

### 2.14 Mass spectrometry and liquid chromatography

A Thermo Scientific Q-Exactive hybrid Quadropole-Orbitrap mass spectrometer (Thermo Fisher, San Jose, CA) was utilized for all liquid chromatography-mass spectrometry (LC/MS) measurements. A Thermo Scientific Vanquish Ultra-high performance Liquid Chromatograph system (Thermo Fisher, San Jose, CA) was utilized for the separation of the digestion samples with subsequent mass measurement. Liquid chromatography settings are as follows: separation of peptides in digestion samples was conducted utilizing a Phenomenex Aeris Peptide XB-C18 analytical column with an Agilent Eclipse XDB-C18 guard. A buffer system consisting of 0.1% formic acid in ultrapure water (mobile phase A) and 0.1% formic acid in acetonitrile (mobile phase B) was also used. Each digestion sample was injected in triplicate and separation was achieved over a 60-minute separation utilizing the following gradient elution: Initial conditions 5.0% B, 0.0 min to 45.0 min linear gradient to 65.0% B, 45 min to 50 min linear gradient to 95.0% B, 55 min 95.0% B, 55 min to 55.1 min linear gradient to 5.0% B, 55.1 min to 60.0 min 5% B at a flow rate of 250 µL min^-1^. The column temperature during the separation was maintained at 40°C with an autosampler temperature of 4°C.

Mass spectrometer operational parameters were optimized utilizing the Tune software for a flow rate of 250 µL min^-1^. Experiments were conducted in positive ion mode at +4.5 kV utilizing a mass-to-charge (m/z) scan range of 275 – 2000 (1 microscan). The instrument was operated at a resolving power of 70,000 for all full scan m/z measurements with an AGC target setting of 1 x 10^6^ and an S-lens voltage of 50 V. The inlet capillary temperature was maintained at 250°C with the following nitrogen gas flow rates utilized for the HESI source: sheath gas flow rate was set to 25 arbitrary units (arb), the auxiliary gas flow rate was set to 12 arb with auxiliary gas heater maintained at 112.5 °C. MS^2^ scans were obtained for the top 40 peaks at a resolution setting of 17,500 with an AGC target of 1 x 10^5^ and an isolation window of ± 4.0 *m/z*. Stepped normalized collision energy (NCE) was set to 28 eV with a dynamic exclusion time of 10.0 seconds. Raw data files from the mass spectrometer were utilized to identify peptides present within the digestion samples utilizing the Proteome Discoverer software suite (Thermo Fisher). Dataset features were identified using exact mass comparisons for retention time (*t_R_*) resolved mass spectra with default parameters for MS^2^ spectra utilizing Orbitrap *m/z* detection. Peptide sequence matches were detected in positive ion mode ranging from [M+H]^+^ to [M + 6H]^+6^ ions with sequence lengths ranging from 6 to 25. Proteins were identified utilizing the ProteinProspector software (UCSF) and uploading known peptide masses into the MS Fit database.

### 2.15 Statistical analysis

All data were analyzed using GraphPad Prism software. P-values and tests used to determine statistical significance can be found within figure legends.

## 3 Results

### 3.1 The mammalian erythrocyte protein, Band 3, is required for *F. tularensis* invasion

We hypothesized that *F. tularensis* uses red blood cell (RBC) surface protein Band 3 as a receptor to invade erythrocytes. This hypothesis is based on several lines of circumstantial evidence. First, a study by Schmitt et al. found that treating human RBCs with *P. guttatus* (blue-bellied black snake) venom inhibits *F. tularensis* LVS invasion (Schmitt et al., 2017). The venom binds to Band 3 on the surface of RBCs and disrupts the RBC’s spectrin filament architecture, suggesting that either Band 3, Spectrin, or both are involved.

**Figure 1:**
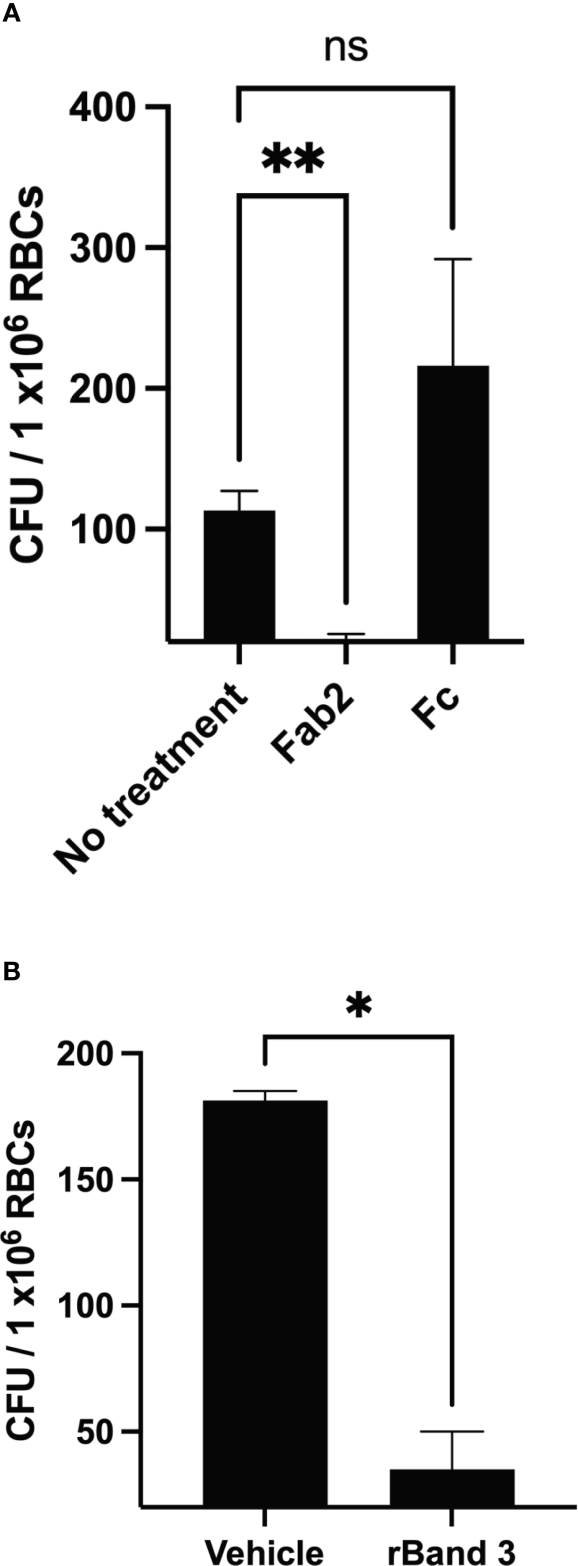
Human erythrocyte protein Band 3 plays a role in erythrocyte invasion by *F. tularensis*. Gentamicin protection assays were used to determine whether *F. tularensis* invaded human RBCs in the presence of anti-Band 3 Fab2 fragments (A) or recombinant Band 3 (B). Each graph represents the combined results of at least three independent experiments from three different blood donors. **(A)** Erythrocytes were treated with anti-Band 3 Fab2 fragment (Fab2, 2 mg/ml) or the isolated constant region of the anti-Band 3 antibody (Fc, 1.1 mg/ml). Data were analyzed via a one way ANOVA with a Dunnett’s *post-hoc* test; P = 0.0047, No treatment vs. Fab2, P = 0.0059**; No treatment vs. FC, P = 0.4373, NS). **(B)** *F. tularensis* LVS cultures were treated with recombinant Band 3, rBand 3 (10 μg/ml) or a vehicle control. Data were analyzed by an unpaired t test; P = 0.0110*. Data represent the mean +/- SD.

To determine the role of Band 3 in invasion of RBCs by *F. tularensis*, we used similar methodologies to those used to evaluate the role of this host protein during erythrocyte invasion by other microbes (Crandall et al., 1993; Hogh et al., 1994; Clough et al., 1995; Goel et al., 2003; Deng et al., 2012; Baldwin et al., 2014; Almukadi et al., 2019). Firstly, this host membrane protein was physically blocked using anti-Band 3 Fab2 fragments. Fab2 fragments were used because RBC invasion requires intact human serum and therefore whole antibodies against Band 3 would have activated complement and lysed the targeted erythrocytes (Horzempa et al., 2011). Here, RBCs were incubated with anti-Band 3 Fab2 fragments for one hour prior to the addition of *F. tularensis* LVS bacteria. Blocking Band 3 in this fashion inhibited erythrocyte invasion by *F. tularensis* (**Figure 1A**). However, incubation with Fc portions of the anti-Band 3 antibody had no effect on RBC invasion (**Figure 1A**). To validate this finding, *F. tularensis* LVS was incubated with rBand 3 for one hour before adding the erythrocytes to evaluate invasion (**Figure 1B**). Treatment with rBand 3 (10 μg/ml) diminished invasion, as would be expected in a competitive inhibition of bacterial binding with the erythrocyte Band 3 (**Figure 1B**). A similar reduction in erythrocyte invasion was observed when rBand 3 was included at 5 μg/ml and 15 μg/ml (data not shown). These findings support the hypothesis that the erythrocyte Band 3 protein plays a role in RBC invasion by *F. tularensis*.

### 3.2 Glycophorin a inhibits erythrocyte invasion by *F. tularensis*

Band 3 interacts with several integral membrane proteins such as Glycophorin A (GPA) and ankyrin to form complexes with the spectrin cytoskeleton (van den Akker et al., 2010). Therefore, given the data that Band 3 may contribute to erythrocyte invasion, it is possible that other proteins that complex with this host protein are important for red blood cell invasion by *F. tularensis*. Furthermore, GPA has been identified as a receptor for *Babesia divergens* and *Plasmodium falciparum* facilitating red blood cell invasion (Lobo, 2005). Therefore, we sought to determine if Glycophorin A plays a role in erythrocyte invasion by *F. tularensis*.

**Figure 2:**
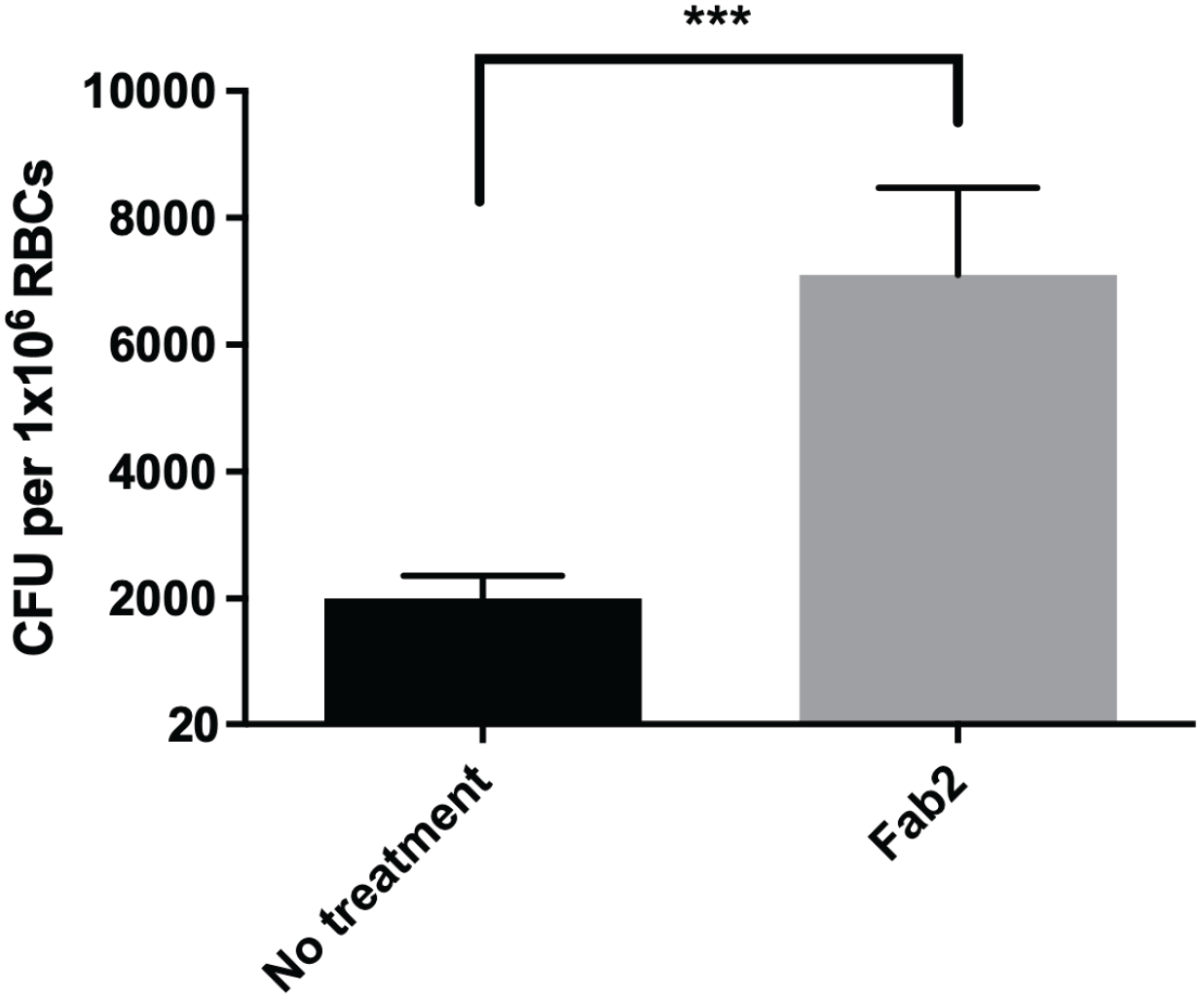
Inhibition of Glycophorin A on human erythrocytes by Fab2 fragments results in increased erythrocyte invasion by *F. tularensis* LVS. Human erythrocytes were treated with anti-Glycophorin A (GPA) Fab2 fragments for 1 hour. Cells were washed and then incubated with *F. tularensis* LVS at an MOI of 12.5 for at least 2 hours. Following incubation, cells were treated with gentamicin for 40 minutes, washed, lysed, then plated on chocolate agar to determine CFU. The CFU ml^-1^ per 1x10^6^ Red Blood Cells (RBCs) was significantly higher in cells treated with anti-Glycophorin A Fab2 fragments compared to those that were untreated (***p = .0025 determined by unpaired t-test with Welch’s correction). Data shown represent the mean +/- SD of a representative experiment that was repeated using blood from two different blood donors.

To study the potential role of GPA in *F. tularensis* erythrocyte invasion, we inhibited interactions with this host protein using Fab2 fragments and carried out a gentamicin protection assay. Surprisingly, erythrocytes treated with anti-GPA Fab2 fragments resulted in a significant increase in invasion by *F. tularensis* LVS compared to the untreated erythrocytes (**Figure 2**) suggesting that this host protein may be antagonizing bacterial entry.

### 3.3 Ankyrin-1 contributes to erythrocyte invasion by *F. tularensis* LVS

**Figure 3:**
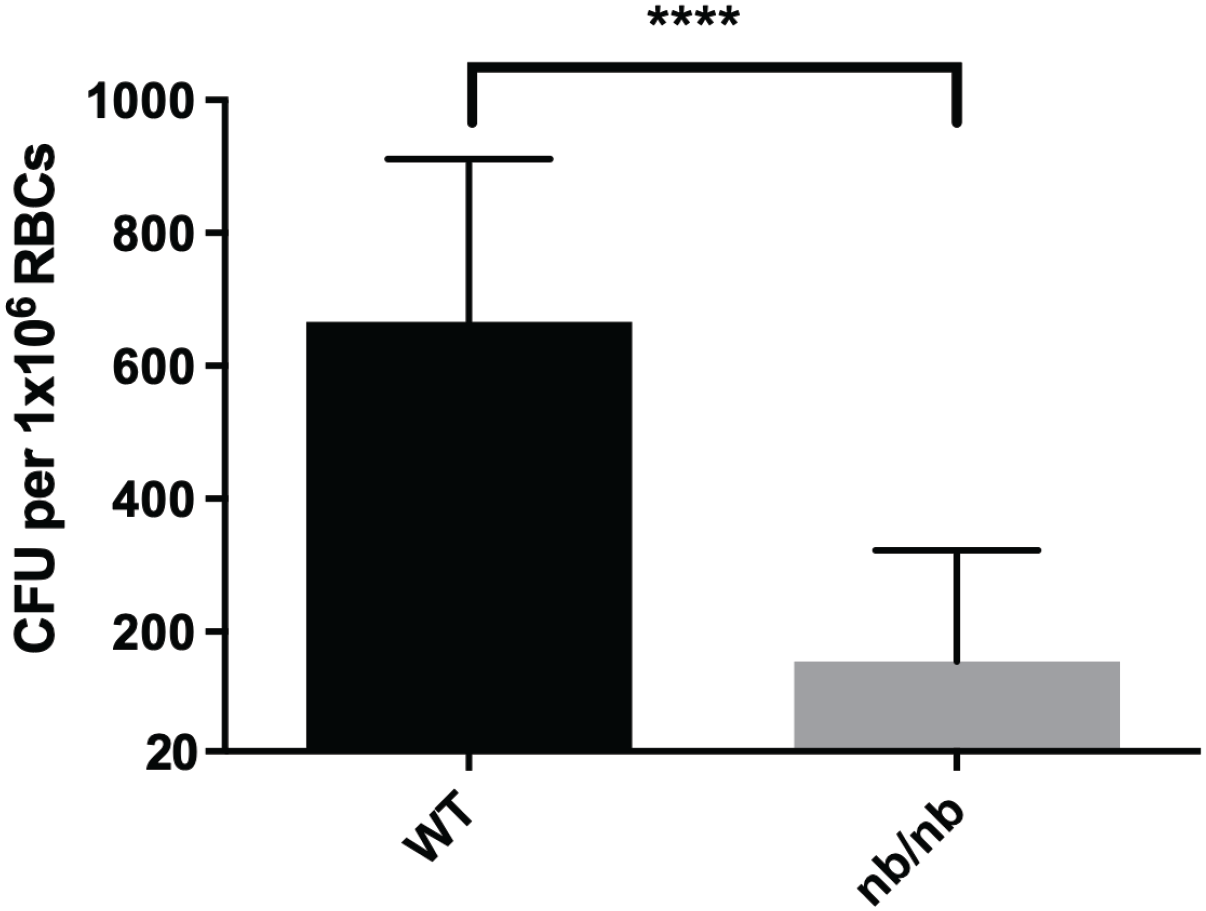
Ankyrin contributes to erythrocyte invasion by *F. tularensis.* Ankyrin-deficient murine erythrocytes (*nb/nb)* and wild type murine erythrocytes were incubated with *F. tularensis* LVS at an MOI of 12.5 for at least two hours. Cells were then treated with gentamicin (25 ug/ml) for 40 minutes, washed once with PBS, then lysed using 0.02% sodium dodecyl sulfate then plated onto chocolate media to determine CFU. The CFU ml^-1^ per 1x10^6^ Red Blood Cells (RBCs) was significantly lower in Ankyrin-deficient (*nb/nb)* erythrocytes compared to wild type (WT) erythrocytes (****p <0.0001 determined by unpaired t-test with Welch’s correction). Data shown represent the mean +/- SD of a representative experiment from two biological replicates.

Engulfment of bacteria or invasion of host cells is typically mediated by the host cytoskeleton (Adam et al., 1995; Clemens et al., 2005). Therefore, *F. tularensis* likely manipulates the erythrocyte spectrin cytoskeleton to invade these host cells. This hypothesis is supported by the previous finding that spectrin impairment inhibits red blood cell invasion by *F. tularensis* (Schmitt et al., 2017). Band 3 is anchored to the spectrin cytoskeleton in two distinct complexes: the Band 3/Ankyrin complex and the 4.1R complex (Salomao et al., 2008; Mankelow et al., 2012). In the 4.1R complex, Band 3 is attached to the spectrin cytoskeleton via small actin bundles and the 4.1 protein. Previous data indicated that actin was dispensable for erythrocyte invasion (Schmitt et al., 2017). In the other complex, Band 3 interacts with Ankryin-1 and other accessory proteins to attach to the spectrin cytoskeleton. Given the potential role of Band 3 and Spectrin, here we sought to investigate if Ankyrin-1 is required for erythrocyte invasion by *F. tularensis.* Erythrocytes were isolated from the whole blood of Ankyrin-1-deficient mice (*nb/nb)* or isogenic wild-type mice and were subjected to a gentamicin protection assay. Here, Ankyrin-deficient erythrocytes (*nb/nb)* exhibited decreased invasion by *F. tularensis* compared to red blood cells isolated from wild-type mice (**Figure 3**). We did not observe a noticeable loss in the number of erythrocytes from the (*nb/nb)* mice compared to the wild-type animals during the duration of these *in vitro* studies, suggesting that cell lysis was not likely responsible for the disparity in erythrocyte invasion observed here (data not shown). These data suggest that erythroid Ankyrin-1 is required for red blood cell invasion by *F. tularensis*.

### 3.4 Identification of *F. tularensis* proteins that potentially interact with the cytoplasmic domain of Band 3

**Figure 4:**
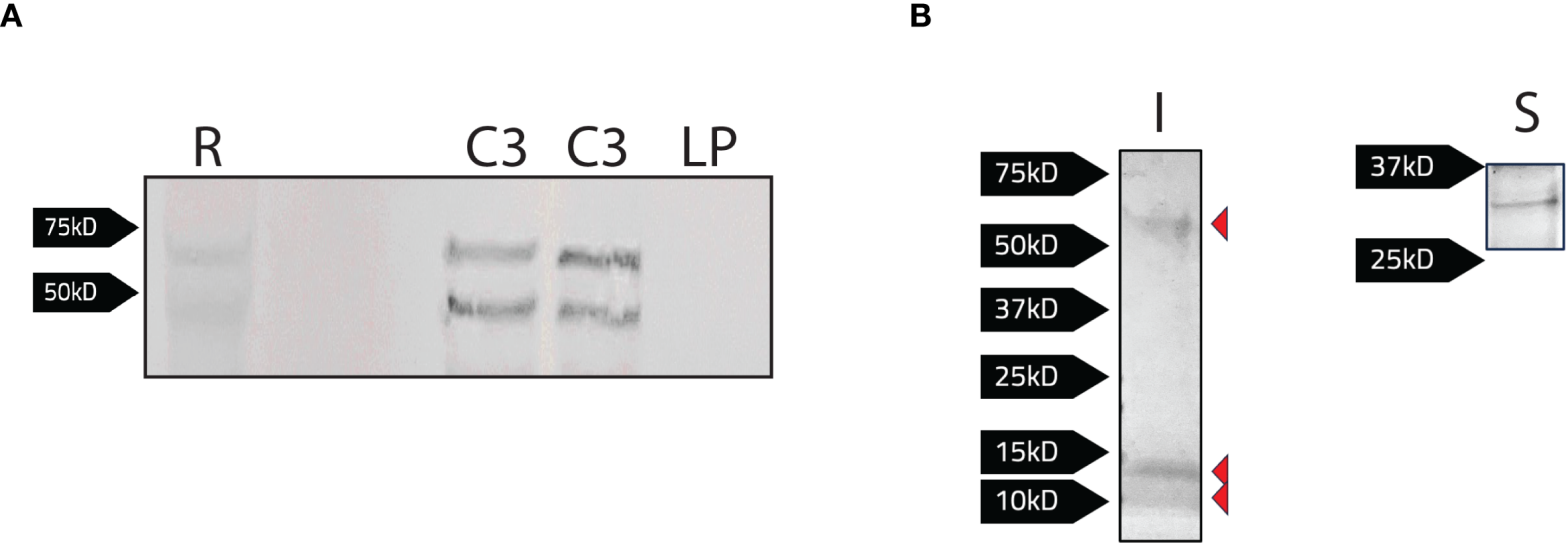
Detection of bacterial proteins interacting with the recombinant cytoplasmic domain of Band 3 expressed in *F. tularensis* LVS. **(A)** Protein levels were normalized via a Bradford Assay and equivalent protein levels of LVS/pCDB3 (C3; 2 bacterial isolates shown here) and LVS/pKHEG (LP) and red blood cell lysates (R) were subject to SDS-PAGE and western blot analysis. The blot was probed with a rabbit anti-Band 3 antibody [1:1000] and then goat anti-rabbit alkaline phosphatase antibody [1:1000]. Expression of the cytoplasmic domain is present in LVS/pCDB3. **(B)** Magnetic agarose beads conjugated with a rabbit anti-Band 3 antibody, (100μg) were incubated with whole cell lysates of LVS/pCBD3. Samples were eluted following the addition of 2x Laemmli buffer with 5% 2-mercaptoethanol to bead complexes and boiled at 95°C for 5 minutes. Samples were then subject to SDS-PAGE. Imperial stain **(I)** and silver stain (S) revealed four potential *F. tularensis* LVS proteins of 10, 15, 30, and 60kD interacting with Band 3.

Given the potential role of Band 3 in RBC invasion, we sought to identify *F. tularensis* bacterial proteins that interact with this host protein to further elucidate the mechanism of RBC entry. Further, given that Ankyrin-1 is required for invasion, and that this protein binds to the cytoplasmic domain of Band 3, we reasoned that this domain may be a target of *F. tularensis* effectors. To identify potential binding partners between *F. tularensis* and the cytoplasmic domain of Band 3, we recombinantly expressed the cytoplasmic domain of Band 3 (CBD3) (encoded in pCDB3) in *F. tularensis* LVS. Western blotting was used to confirm expression of the recombinant Band 3 protein in LVS (**Figure 4A**). When probed with an anti-Band 3 polyclonal antibody, two bands were observed at approximately 45 kD and 70 kD for LVS/pCDB3, which matched similar banding patterns observed with the red blood cell control (**Figure 4A**). No bands were observed in LVS bacteria harboring the parent vector (pKHEG), indicating the bands observed in LVS/pCDB3 were due to the expression of the cytoplasmic domain of Band 3.

Next, co-immunoprecipitation using the recombinant cytoplasmic domain of Band 3 as “the bait” was performed to identify any potential *F. tularensis* binding partners from bacterial lysates. Here, an anti-Band 3 polyclonal antibody was conjugated to magnetic agarose beads and co-incubated with LVS/pCDB3 lysates for at least 2 hours. Beads were then washed twice with PBS, and protein complexes were eluted by boiling beads in 2x Laemmli buffer supplemented with 5% 2-mercaptoethanol and boiling at 95°C for 5 minutes. Following sample elution and SDS-PAGE, imperial staining and silver staining revealed four potential *F. tularensis* interacting proteins of approximately 10, 15, 30, and 60 kD (**Figure 4B**).

**Figure 5:**
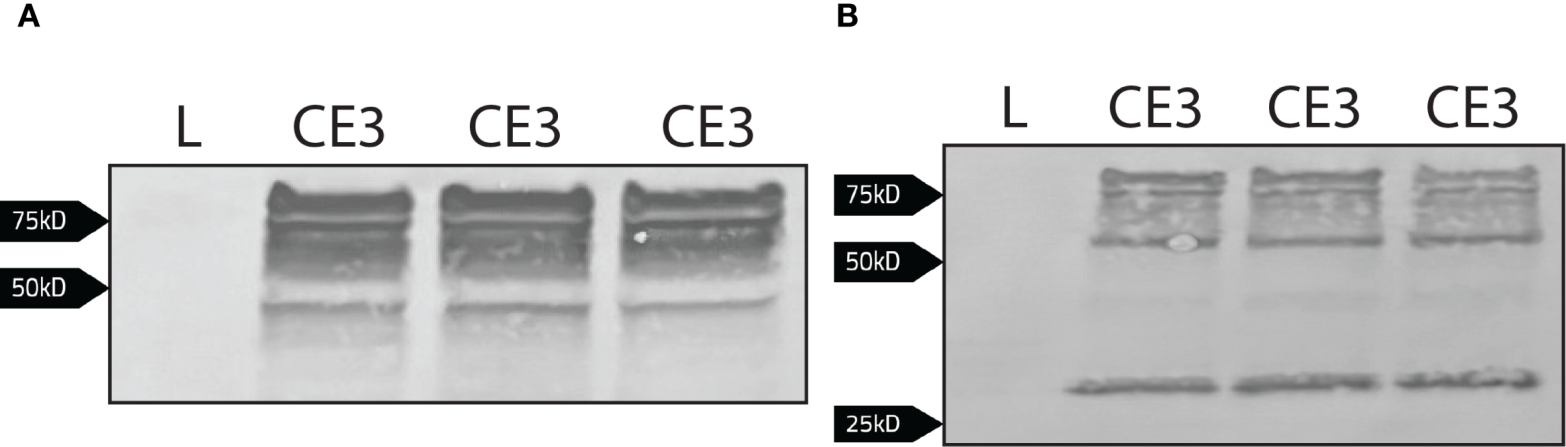
Detection of the cytoplasmic domain of Band 3 linked to emGFP via western blot. Whole cell lysates normalized to the same OD_600_ value of LVS (L) and LVS/pCDB3-emGFP (CE3; three bacterial isolates shown here) were subject to western blot analysis. **(A)** The blot was probed with a rabbit anti-Band 3 antibody [1:1000] and then goat anti-rabbit alkaline phosphatase antibody [1:1000]. Expression of the cytoplasmic domain linked to emGFP is present in LVS/pCDB3-emGFP. **(B)** The blot was probed with a rabbit anti-emGFP antibody [1:1000] and then goat anti-rabbit alkaline phosphatase antibody [1:1000].

The polyclonal antibody used to immobilize the cytoplasmic portion of Band 3 could have potentially interfered with the binding of bacterial proteins in the co-immunoprecipitation depicted in **Figure 4B**. Therefore, as an alternative approach, coding sequence for the cytoplasmic domain of Band 3 (CDB3) was fused to emerald green fluorescent protein (CDB3-EmGFP; encoded in pCDB3-emGFP) and this chimeric protein was recombinantly expressed in *F. tularensis* LVS. In this iteration, the EmGFP domain was targeted in a co-immunoprecipitation assay to identify *F. tularensis* LVS protein binding partners to the cytoplasmic domain of Band 3. Western blotting was used to determine expression of the CDB3-EmGFP fusion. When probed with an anti-Band 3 polyclonal antibody, bands were observed between from ~50 kD to above 75 kD with additional bands below the 50 kD marker for *F. tularensis* LVS/pCDB3-emGFP, while lysates from *F. tularensis* LVS alone produced no detectible bands (**Figure 5A**). When probed with an anti-GFP monoclonal antibody, bands were also observed above 50 kD to above 75 kD as well as a band near 27 kD (**Figure 5B**). However, *F. tularensis* LVS alone again produced no detectible bands (**Figure 5B**). These data indicate that the observed bands were due to the expression of the cytoplasmic domain of Band 3 linked to emGFP.

We next sought to confirm expression of CDB3-emGFP using fluorescence microscopy. *F. tularensis* LVS cells producing recombinant CBD3-emGFP were grown in Chamberlain’s Defined Medium (CDM) to stationary phase at 37 °C with agitation, then added to 1% agarose pads in PBS to visualize the bacteria. In LVS/pCDB3-emGFP, we observed robust fluorescence in cells expressing cdb3-emgfp, compared to wild type bacteria which elicited no detectible fluorescence (**Supplementary Figure S1** in the **Supplementary Material**). Fluorescence was exhibited uniformly throughout the cytoplasm of LVS/pCDB3-emGFP, suggesting expression of CDB3-emGFP does not result in localization of the resulting fusion protein to a specific bacterial site nor did expression of this recombinant protein produce evidence of inclusion bodies which suggests that this protein did not misfold.

**Figure 6:**
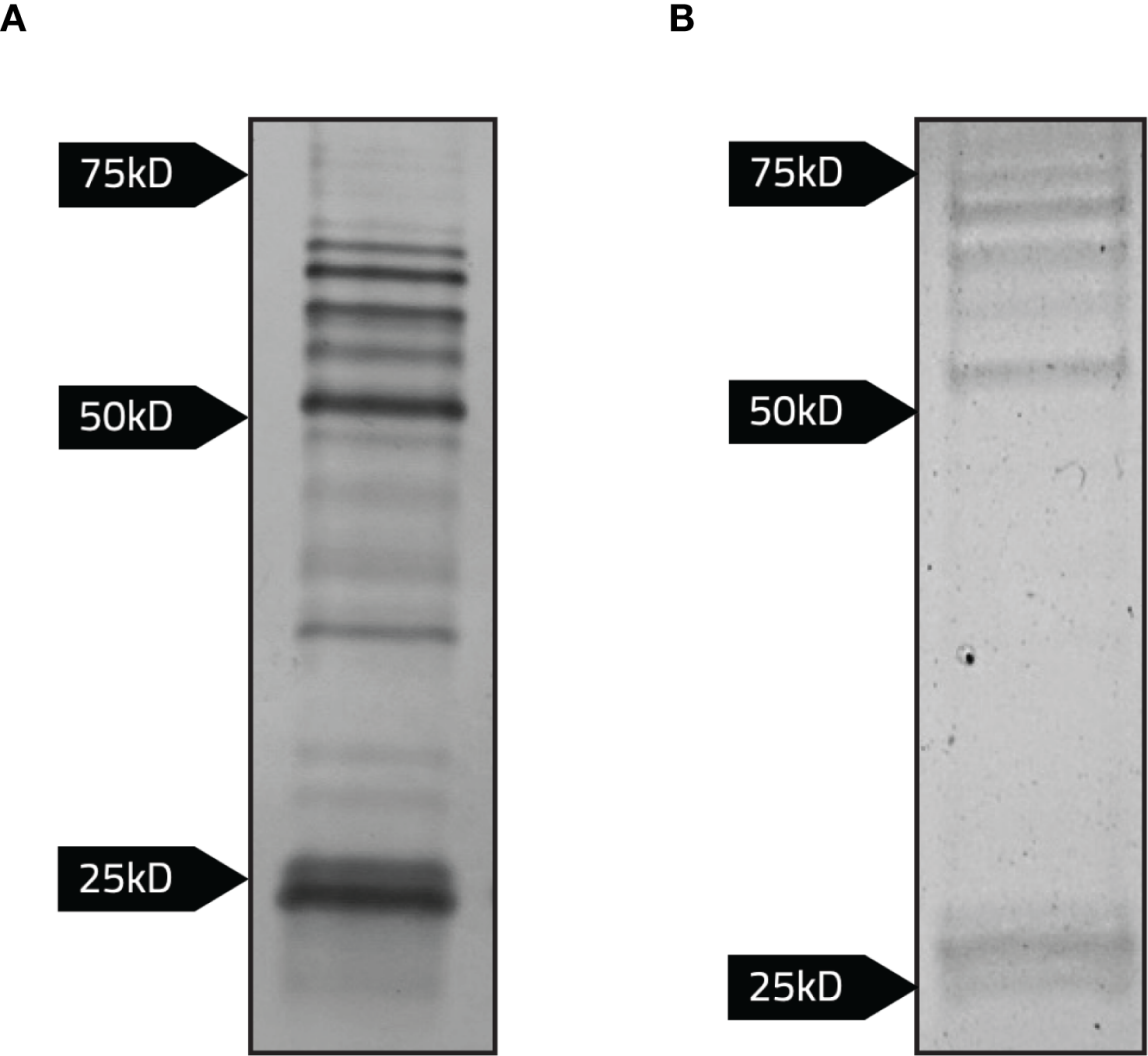
Detection of bacterial proteins interacting with the cytoplasmic domain of Band 3 linked to emGFP. Magnetic agarose beads conjugated to anti-GFP nanobodies were incubated with whole lysates normalized to the same OD_600_ of LVS, LVS/pCDB3-emGFP, and LVS/pKHEG. Samples were eluted following the addition of 2x Laemmli buffer with 5% 2-mercaptoethanol to bead complexes and boiled at 95°C for 5 minutes. Samples were then subject to SDS-PAGE. **(A)** Silver staining revealed potential LVS proteins interacting with the recombinant cytoplasmic domain of Band 3. **(B)** Imperial staining was performed to identify LVS proteins binding to the recombinant cytoplasmic domain of Band 3.

Next, potential CBD3-EmGFP protein complexes were isolated using magnetic beads conjugated to anti-GFP nanobodies (ChromoTek GFP-Trap). These beads were co-incubated with LVS/pCDB3-emGFP lysates for at least 2 hours at 37 °C. Samples were eluted and analyzed using SDS-PAGE. Proteins were visualized by staining using imperial (**Figure 6A**) and silver stains (**Figure 6B**). Staining revealed potential *F. tularensis* bacterial proteins binding to the cytoplasmic domain of Band 3 (**Figure 6**). To ensure that these bacterial proteins were not directly binding to EmGFP, we incubated magnetic beads conjugated to anti-GFP nanobodies with bacterial lysates containing the parent vector expressing only EmGFP (LVS/pKHEG). No bands were observed following SDS-PAGE and staining of lysates from this control strain, indicating the bacterial proteins observed were interacting with the cytoplasmic domain of Band 3 and not the EmGFP portion of this chimeric protein (data not shown).

To identify the *F. tularensis* LVS bacterial proteins potentially interacting with the cytoplasmic domain of Band 3, bands of interest were excised from the stained gels and subject to in-gel proteomic digestion. This digested material was analyzed using liquid chromatography-mass spectrometry to identify peptide masses. These peptide masses were subsequently analyzed using the ProteinProspector software MS-Fit database (UCSF) by crossing samples with known *F. tularensis* proteins. The Molecular Weight Search Engine (MOWSE) score was used to determine how well observed peptide masses match with peptide masses from known proteins (Damodaran et al., 2007). A MOWSE score higher than 50 indicates a statistically significant match between observed peptide masses and peptide masses of a particular protein (**Table 2**). Some notable proteins that were identified that fulfill this criterion include the outer membrane vesicle-associated lipase protein, FtlA (**Table 2**). FtlA has been found to contribute to attachment and entry into host cells and pathogenesis in a mouse model of tularemia (Chen et al., 2017). A conserved outer membrane protein of *F. tularensis*, known as LpnA (Tul4) was also identified (**Table 2**). This protein has been shown to stimulate toll-like receptor 2 (TLR2) in human macrophages but does not affect *F. tularensis* virulence in mice (Forestal et al., 2008). The glycine cleavage protein T (GcvT), which is a part of the glycine cleavage system in *F. tularensis* was identified as a potential interactor with Band 3 (**Table 2**). This protein has been shown to contribute to pathogenesis in murine models as well as intracellular replication in serine limiting environments (**Table 2**) (Brown et al., 2014).

**Table 2:** Identification of *F. tularensis* LVS proteins potentially interacting with the recombinant cytoplasmic domain of Band 3.

Protein	Gene name	Gene number	Protein description	Strain	MOWSE score	Excised band size
GltX	*gltX*	FTL_0218	Glutamyl-tRNA ligase	LVS/pCDB3-emGFP	841	55kD
FtlA	*ftlA*	FTL_0430	Outer membrane vesicle-associated lipase	LVS/pCDB3-emGFP	518	55kD
KatG	*katG*	FTL_1504	Catalase-Peroxidase	LVS/pCDB3-emGFP	515	80kD
GcvT	*gcvT*	FTL_0447	Aminomethyltransferase	LVS/pCDB3-emGFP	416	30kD, 40kD,
LpnA	*lpnA*	FTL_0421	17kDa major membrane protein	LVS/pCDB3-emGFP	340	15kD
CoaE	*coaE*	FTL_0307	Dephospho-CoA kinase	LVS/pCDB3-emGFP	265	43kD, 60kD
_	*_*	FTL_0548	dITP/XTP pyrophosphatase	LVS/pCDB3-emGFP	184	70kD
MetG	*metG*	FTL_0444	Methionyl-tRNA synthetase	LVS/pCDB3	175	10kD
_	*_*	FTN_1103	Bacterial lipoprotein	LVS/pCDB3	164	30kD
UvrC	*uvrC*	FTL_1448	Exinuclease ABC, subunit C	LVS/pCDB3-emGFP	161	40kD, 43kD, 50kD
MnmE	*mnmE*	FTL_1177	GTPase of unknown function	LVS/pCDB3-emGFP	110	40kD
TrpD	*trpD*	FTN_1776	Anthranilate phosphoribosyltransferase	LVS/pCDB3-emGFP	105	25kD
HslU	*hslU*	FTL_0964	ATP-dependent protease, ATPase subunit	LVS/pCDB3-emGFP	66.1	70kD
HemH	*hemH*	FTL_0821	Ferrochelatase	LVS/pCDB3-emGFP	57.0	60kD

**Figure 7:**
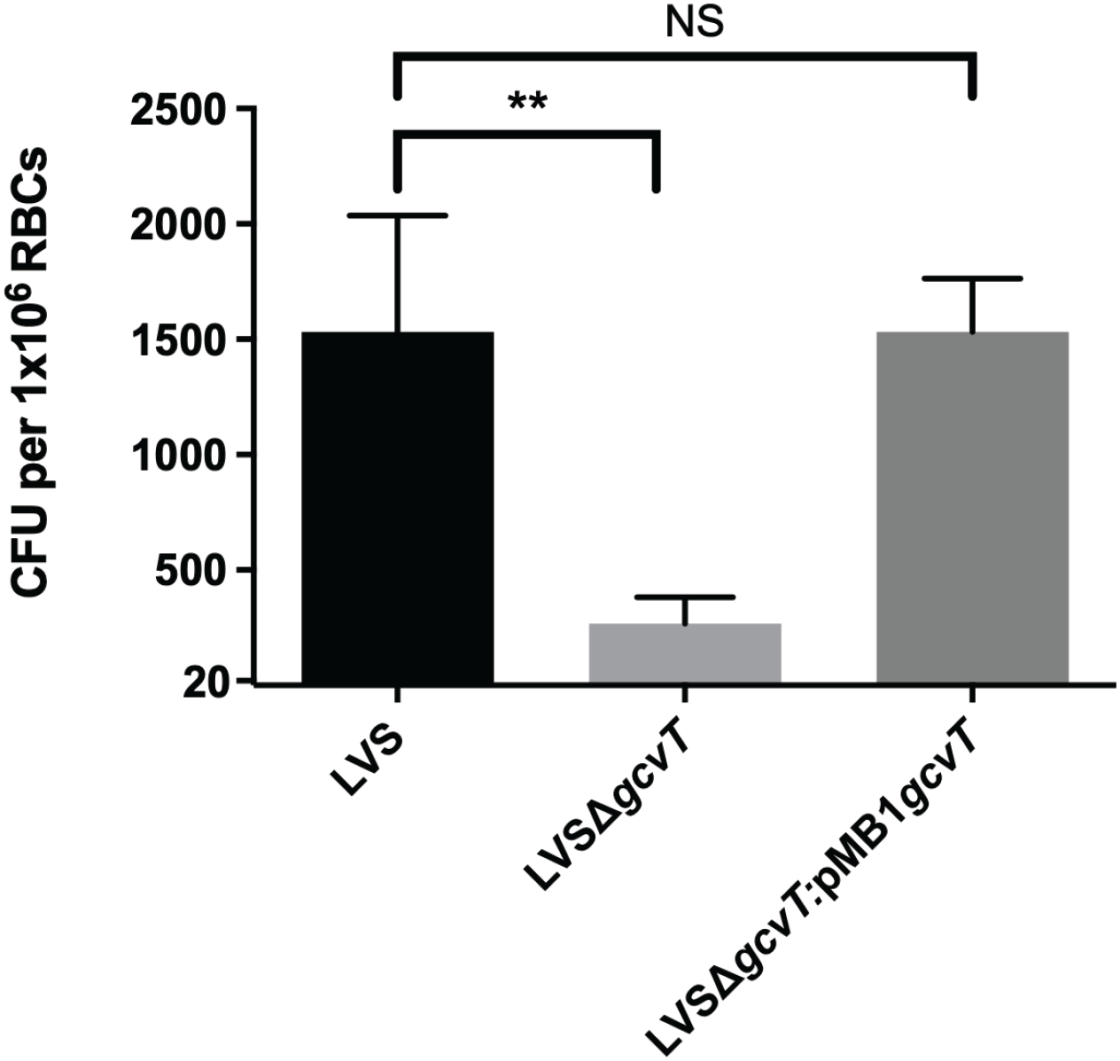
Deletion of *gcvT* results in decreased erythrocyte invasion by *F. tularensis* LVS. Human erythrocytes were incubated with either *F. tularensis* LVS or *F. tularensis* LVSΔ*gcvT* at an MOI of 12.5 for at least two hours. Cells were then treated with gentamicin (25 ug/ml) for 40 minutes, washed once with PBS, then lysed using 0.02% sodium dodecyl sulfate then plated onto chocolate media to determine CFU. The CFU ml^-1^ per 1x10^6^ Red Blood Cells (RBCs) was significantly lower for LVSΔ*gcvT* compared to wild type (**p = .0036 determined by 1-way ANOVA with Dunnett’s multiple comparisons test). Introduction of *gcvT* into LVSΔ*gcvT* via complementation vector restores invasion. Data shown represent the mean +/- SD of a representative experiment that was repeated using blood from two different blood donors.

Given the previous research conducted on the role of GcvT in *F. tularensis* pathogenesis, as well as serendipitously having a GcvT deletion mutant on hand, we sought to investigate the potential role of GcvT in erythrocyte invasion. To determine if GcvT is important for erythrocyte invasion, we performed a gentamicin protection assay using the *F. tularensis* LVS *gcvT* deletion mutant. Here, human erythrocytes were isolated from whole blood and incubated with the indicated *F. tularensis* strains at an MOI ~ 12.5 for 2 hours (**Figure 7**). Following incubation, cells were treated with gentamicin to kill extracellular bacteria, washed with PBS, and then lysed and plated to enumerate CFU (**Figure 7**). This assay indicated that the *gcvT* strain exhibited a significant decrease in erythrocyte invasion compared to the wild type bacteria (**Figure 7**). Complementation of *gcvT* in the *F. tularensis gcvT* mutant rescued invasion to wild-type levels indicating that the observed decrease in erythrocyte invasion by the mutant was not due to a polar or pleiotropic effect. Moreover, disk diffusion assays indicated that the *gcvT* mutant exhibited a similar level of sensitivity to both gentamicin and SDS compared the wild type strain (data not shown). Therefore, the observed decrease in mutant bacteria surviving the gentamicin protection assay was unlikely due to an artifact of this experimental methodology (data not shown). These data suggest that GcvT is important for erythrocyte invasion by *F. tularensis* likely through an interaction with the cytoplasmic domain of Band 3.

### 3.5 Erythrocyte spectrin (alpha chain) interacts with the *F. tularensis* effector protein, PdpC

**Figure 8:**
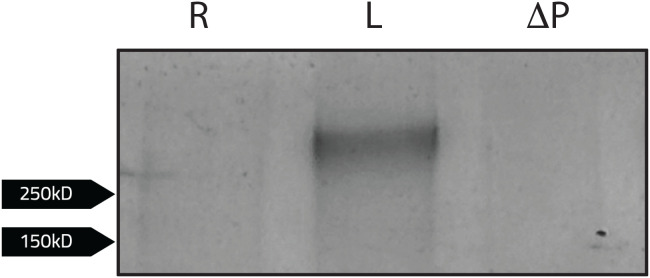
Human erythrocyte proteins interacting with PdpC. Magnetic agarose beads conjugated to an anti-PdpC rabbit antibody (100ug) were incubated with *F. tularensis* LVS (L) or *F. tularensis* LVS *pdpC*-null (ΔP) lysates for at least 2 hours. Beads incubated with bacterial lysates were washed than incubated with lysed human erythrocytes diluted to a desired concentration (1 x 10^7^ cells/ml) in red blood cell media for 2 hours. Samples were eluted following the addition of 2x Laemmli buffer with 5% 2-mercaptoethanol to bead complexes and boiled at 95°C for 5 minutes. Samples were then subject to SDS-PAGE. Following silver stain analysis, a single band was observed when erythrocytes were incubated with *F. tularensis* LVS (L). No bands were observed when LVS was incubated without erythrocytes lysates (R).

Because PdpC is required for attachment to and invasion of red blood cells, we hypothesized that identifying erythrocyte proteins that interact with PdpC could provide insight into the mechanism of red blood cell invasion (Cantlay et al., 2022). To identify potential erythrocyte binding partners of PdpC, a co-immunoprecipitation assay was performed. Magnetic agarose beads were conjugated to an anti-PdpC polyclonal antibody and co-incubated with *F. tularensis* LVS lysates for at least 2 hours. Following incubation, isolated lysed human erythrocytes diluted to a desired concentration (1 x 10^7^ cells/ml) were added to the beads and incubated at 37 °C for another two hours. As a control, erythrocyte proteins were also added to antibody-conjugated beads incubated with lysates from a *F. tularensis* mutant strain lacking PdpC to assess potential non-specific binding. The beads were then washed twice with PBS, and the bound protein complexes were eluted by boiling beads in 2x Laemmli buffer supplemented with 5% 2-mercaptoethanol. Samples were analyzed via SDS-PAGE, and proteins were visualized by silver stain. A single band was observed above 250 kD but was not observed in our LVS *pdpC*-null mutant (**Figure 8**). To confirm the band observed was not PdpC, a western blot was conducted using an anti-PdpC polyclonal antibody (data not shown). PdpC was not detected, indicating the band observed was an erythrocyte protein.

To identify the potential erythrocyte protein interacting with PdpC, the single band was excised from the gel and subject to in-gel proteomic digestion to purify the protein. The sample was then analyzed using liquid chromatography-mass spectrometry to identify peptide masses. Peptide masses were subsequently analyzed using the ProteinProspector software MS-Fit database (UCSF) by cross referencing with peptide masses of known human erythrocyte proteins. Human erythrocyte proteins that most closely fit sample peptide masses (MOWSE score >50) were then identified (**Table 3**). The erythrocyte protein identified to potentially interact with PdpC was Spectrin (alpha chain) (MOWSE score of 3086; **Table 3**). This finding supports previous data suggesting the involvement of spectrin in erythrocyte invasion (Schmitt et al., 2017). This result also indicates that PdpC interacts with the major erythrocyte cytoskeletal protein, Spectrin (alpha chain), potentially mediating erythrocyte invasion through this interaction.

**Table 3:** Identification of the potential interactions between PdpC and human erythrocyte proteins.

Protein	Protein code	Gene name	MOWSE score
Spectrin, alpha chain	SPTA1	*SPTA1*	3086

## 4 Discussion

**Figure 9:**
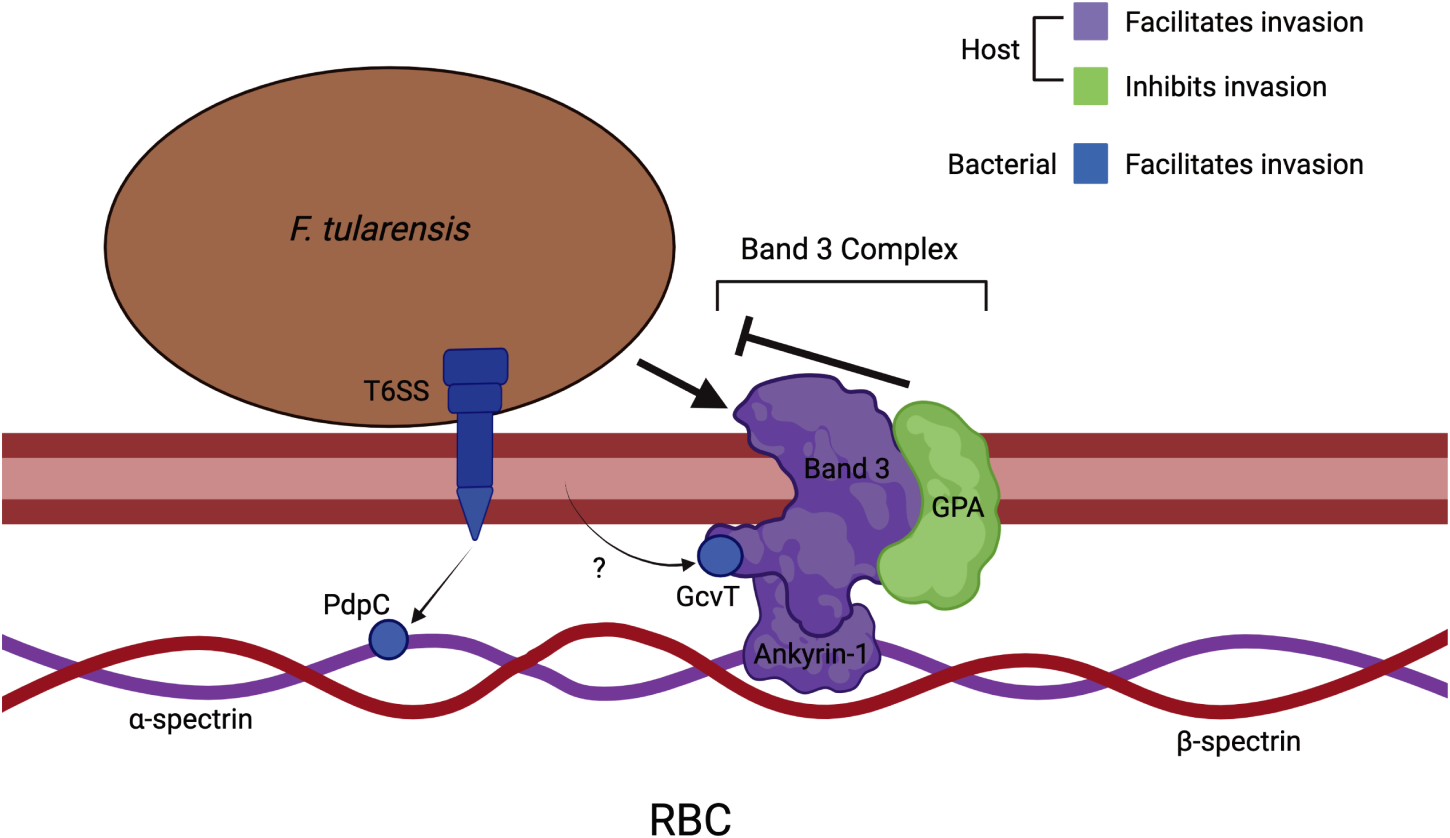
Host-pathogen interactions that mediate erythrocyte invasion by *F. tularensis.* Bacterial interaction with the erythrocyte membrane protein, Band3 of the Band3 complex, initiates invasion while GPA of this complex inhibits this phenomenon. Additionally, Ankyrin-1 – the component of the Band 3 complex that links the erythrocyte membrane to the underlying cytoskeleton – is important in erythrocyte invasion. The bacterial Type VI Secretion System (T6SS) translocates PdpC across the erythrocyte membrane. Subsequently, PdpC interacts with spectrin (α-chain) of the erythrocyte cytoskeleton, and we propose that this interaction facilitates invasion. Additionally, GctA has been shown to interact with the cytoplasmic domain of Band3 and contribute to erythrocyte invasion. This bacterial protein is presumably delivered to the erythrocyte cytosol by a mechanism that has not yet been elucidated. This image was created with BioRender.com.

In this study, we identified host-pathogen interactions between *F. tularensis* and mammalian erythrocytes that contribute to erythrocyte invasion. To do so, we investigated the role of the erythrocyte surface protein, Band 3, in *F. tularensis* erythrocyte invasion. Human erythrocytes treated with anti-Band 3 Fab fragments showed a reduction of erythrocyte invasion. Moreover, bacteria treated with recombinantly expressed Band 3 protein exhibited a reduced capacity to invade RBCs. These data suggest that Band 3 is required for erythrocyte invasion and could suggest that this molecule is the host receptor used by *F. tularensis* prior to invasion (**Figure 9**).

While pathogens that invade red blood cells apparently utilize distinct bacterial mechanisms to facilitate invasion, the possibility exists that these microorganisms exploit common host molecules and pathways to access the intracellular space. Trw proteins are components of the Type IV Secretion System of *Bartonella birtlesii*, a bacterium isolated from a wild rodent that invades erythrocytes (Bermond et al., 2000). These Trw proteins were found to bind to the membrane glycoprotein, Band 3 of mouse erythrocytes (Deng et al., 2012). Likewise, Band 3 serves as the docking site of the protozoal parasite, *Plasmodium falciparum* (Almukadi et al., 2019). The surface-exposed Band 3 either interacts with the erythrocyte spectrin cytoskeleton through Ankyrin-1 or via a complex of proteins that includes actin (Salomao et al., 2008). Therefore, cytoskeletal rearrangement mediated through interactions with Band 3 could possibly mediate pathogen invasion.

As Glycophorin A is an erythrocyte glycoprotein commonly used by intracellular pathogens to mediate erythrocyte invasion, we sought to investigate the potential role of Glycophorin A (GPA) in erythrocyte invasion (Lobo, 2005; Takabatake et al., 2007; Jaskiewicz et al., 2019). We used anti-GPA Fab2 fragments to physically block Glycophorin A on erythrocytes. Erythrocytes treated with anti-GPA Fab2 fragments exhibited increased invasion by *F. tularensis* compared to wild type. This result was quite surprising, considering the fact Glycophorin A is a key receptor for *Plasmodium falciparum*, *Bartonella* sp. and *Babesia bovis* during erythrocyte invasion and inhibition of GPA resulted in decreased invasion by these pathogens (Buckles and McGinnis Hill, 2000; Lobo, 2005; Jaskiewicz et al., 2019). The enhanced erythrocyte invasion after physically blocking Glycophorin A (**Figure 9**) suggests a conformational change of GPA, potentially revealing new binding domains on Band 3 with which *F. tularensis* can interact. Previous research has also determined binding of anti-GPA antibodies to GPA results in immobilization of GPA and Band 3 on the erythrocyte membrane (Giger et al., 2016). Immobilization of these proteins could give bacteria increased access to binding sites that could facilitate invasion. Immobilization could also alter the erythrocyte membrane, making the membrane more susceptible to bacterial binding and invasion. Glycophorin A could also act to inhibit *F. tularensis* from engaging with the cell membrane during invasion by interacting directly with the bacterium or other erythrocyte cell membrane molecules. By inhibiting Glycophorin A, this could allow for enhanced interactions between the bacterium and the erythrocyte membrane to facilitate invasion. However, more work is required to determine the nature of the antagonism observed by inhibiting GPA as this could be a valuable strategy for preventing erythrocyte invasion.

Following the rationale for our hypothesis, *F. tularensis* could potentially facilitate erythrocyte invasion in a fashion like that of the malaria parasite, *Plasmodium falciparum*. For instance, *P. falciparum* can utilize two separate pathways for erythrocyte invasion, the sialic acid-dependent and independent pathways (Goel et al., 2003). In the sialic acid-dependent pathway, *P. falciparum* expresses proteins that interact with host sialoglycoprotein Glycophorin A to invade erythrocytes but can switch to the sialic acid-independent pathway, where the parasite expresses a different set of proteins that interact with the glycoprotein Band 3 (Goel et al., 2003). Speculatively, *F. tularensis* could utilize different invasion pathways to interact with different complexes comprised of Band 3 in the erythrocyte membrane, such as the Band 3/Ankyrin complex or the 4.1 R complex, enabling the bacterium to interact with the red blood cell membrane at different sites. If one pathway is compromised due to the masking or inaccessibility of a binding site, *F. tularensis* may still be able to invade using an alternative pathway. If Glycophorin A normally disrupts erythrocyte invasion, then inhibition of this host protein may allow *F. tularensis* to use both pathways, potentially leading to enhanced invasion. This explanation is less likely, as previous research has determined actin, the erythrocyte membrane protein that anchors Band 3 to the spectrin cytoskeleton in the 4.1R complex, is not required for invasion (Schmitt et al., 2017). Additionally, the decreased invasion in the erythrocytes from ank1 deficient mice further argues against this explanation, as ankyrin is only found in the Band 3/Ankyrin complex. Nonetheless, future experimentation should explore whether *F. tularensis* can utilize pathways proposed involving Band 3 to invade erythrocytes.

We also sought to investigate the role of erythrocytic ankyrin-1 in *F. tularensis* erythrocyte invasion. Ankyrin-1 interacts with the cytoplasmic domain of Band 3 to anchor the erythrocyte plasma membrane to the spectrin cytoskeleton (Vani et al., 2024). The connection of the membrane to the cytoskeleton by ankyrin-1 is crucial for membrane integrity and rigidity within erythrocytes (Vani et al., 2024). Previous research showed erythrocytes deficient in ankyrin were resistant to invasion by the malaria parasite (Shear et al., 1991). When incubated with *F. tularensis* LVS, ankyrin-deficient erythrocytes exhibited a significant decrease in invasion compared to wild type erythrocytes, indicating ankyrin-1 contributes to bacterial invasion (**Figure 9**). When studying the effects of ankyrin-deficiency on the erythrocyte membrane, researchers noted a secondary loss of spectrin (Birkenmeier et al., 2003). As previous research showed erythrocytes treated with venom from *P. guttatus* resulted in spectrin impairment and decreased invasion by *F. tularensis*, one could speculate the decreased invasion in ankyrin-deficient erythrocytes was not only due to the loss of ankyrin, but the disorganization of spectrin as well. Impaired invasion could also be due to the increased osmotic fragility of ankyrin-deficient erythrocytes (Rank et al., 2009). Future research should investigate *F. tularensis* proteins that interact with ankyrin to facilitate red blood cell invasion.

To investigate the role of bacterial proteins in erythrocyte invasion, we screened for potential interactions between the cytoplasmic domain of Band 3 and *F. tularensis* proteins. From this screen, we obtained a list of *F. tularensis* proteins including GcvT, the glycine cleavage system (GCS) protein T. This protein contributes to pathogenesis in murine models and intracellular replication in serine limiting environments (Brown et al., 2014). We therefore decided to investigate the role of GcvT in *F. tularensis* erythrocyte invasion using a *F. tularensis gcvT* deletion mutant that we serendipitously had on hand. A significant decrease in invasion was observed in erythrocytes by the *gcvT* strain compared to wild type bacteria, suggesting GcvT contributes to erythrocyte invasion by *F. tularensis* through interactions with the cytoplasmic domain of Band 3 (**Figure 9**).

While the GCS plays an important role in amino acid synthesis within bacteria, GCS proteins in *Mycoplasma* sp. have also been found to interact with host cell kinases to modulate host signaling pathways (Pan et al., 2024). GcvH in *Mycoplasma bovis* interacts with the AMPK-associated kinase, Brsk2, to increase Brsk2 expression and subsequent inhibition of Caspase-3 activation and apoptosis (Pan et al., 2024). A homolog in the GCS of *F. tularensis*, GcvH, shares 24% protein sequence similarity with GcvH from *Mycoplasma bovis*, indicating the potential for functional relatedness between these two proteins. The cytoplasmic domain of Band 3 is considered a substrate for many erythrocyte tyrosine kinases (Ferru et al., 2011). Tyrosine phosphorylation of Band 3 leads to the dissociation of Band 3 from ankyrin and the spectrin cytoskeleton, resulting in membrane deformity and instability (Ferru et al., 2011). The cytoplasmic domain of Band 3 is also a substrate for Caspase-3, where activation of Caspase-3 leads to the cleavage of the cytoplasmic domain and subsequent disruption between Band 3 and ankyrin (Vani et al., 2024). GcvT could potentially interact with erythrocyte tyrosine kinases to increase phosphorylation of the cytoplasmic domain of Band 3, where the subsequent dissociation of Band 3 from ankyrin and loss of membrane integrity could allow for bacterial invasion. Alternatively, GcvT could recruit Caspase-3 to the cytoplasmic domain of Band 3 or activate Caspase-3, resulting in the cleavage of Band 3 and loss of membrane-cytoskeletal anchoring through the disruption between Band 3 and ankyrin. We would be interested in investigating the specific interactions between GcvT and the cytoplasmic domain of Band 3 that could result in the loss of membrane integrity and perhaps facilitate the manipulation of the spectrin cytoskeleton by *F. tularensis* secreted effectors such as PdpC (Schmitt et al., 2017). Pull-down assays using GcvT as the “bait” could help us to identify any “prey” erythrocyte proteins that interact with GcvT during erythrocyte invasion.

As *F. tularensis* utilizes a Type VI Secretion System (T6SS) to secrete proteins into erythrocytes during invasion, future investigations could explore whether GcvT is secreted through this apparatus (Bröms et al., 2010). As GcvT potentially interacts with the cytoplasmic domain of Band 3 in erythrocytes, we predict that GcvT is being secreted into the erythrocyte to facilitate erythrocyte cytoskeletal rearrangement and subsequent bacterial invasion. While GcvT has not yet been identified as a secreted effector of the T6SS or any other bacterial secretion system of *F. tularensis*, future experiments should explore this possibility.

As the T6SS effector protein, PdpC, is required for erythrocyte invasion, we next pursued to identify potential interactions between PdpC and erythrocyte proteins. Following our screening, we identified a potential interaction between PdpC and the alpha chain of Spectrin (**Figure 9**). This interaction supported a previous hypothesis that PdpC manipulates the spectrin cytoskeletal network to facilitate erythrocyte invasion (Schmitt et al., 2017; Cantlay et al., 2022). This result provides a direct link between the involvement of the T6SS and the erythrocyte cytoskeleton and provides the basis to elucidate the mechanism of red blood cell invasion (**Figure 9**). Future work should characterize the PdpC-Spectrin (alpha chain) interaction to provide further insight into the phenomenon of RBC invasion.

In this study, all *in vitro* erythrocyte invasion assays were carried out using the attenuated Type B *F. tularensis* LVS and derived mutants. Previously published *in vitro* assays showed that *F. tularensis* LVS invades erythrocytes to similar levels as the fully virulent Schu S4 strain (Horzempa et al., 2011), suggesting that LVS can be used safely in a biosafety level 2 facility to model erythrocyte invasion. However, future studies should investigate whether fully virulent *F. tularensis* strains utilize similar host-pathogen interactions as the ones elucidated here (**Figure 9**).

In conclusion, this study elucidated host-pathogen interactions that contribute to erythrocyte invasion by *F. tularensis*. The identification of host molecules that are exploited by this pathogenic bacterium will help further our understanding of red blood invasion by *F. tularensis*. In addition, the identification of the novel role of *F. tularensis* GcvT in invasion, and the discovery of a direct link between the T6SS and the erythrocyte cytoskeleton represent substantial steps forward toward our understanding of red blood cell invasion.

## Data Availability

The raw data supporting the conclusions of this article will be made available by the authors, without undue reservation.
