# Supplementary material for: Host-pathogen interactions involved in erythrocyte invasion by Francisella tularensis
Source: Front Cell Infect Microbiol. 2025 Sep 23;15:1664733. doi: 10.3389/fcimb.2025.1664733 (PMC12500703; doi:10.3389/fcimb.2025.1664733)
Supplement: Supplementary file 1 [file DataSheet1.pdf]

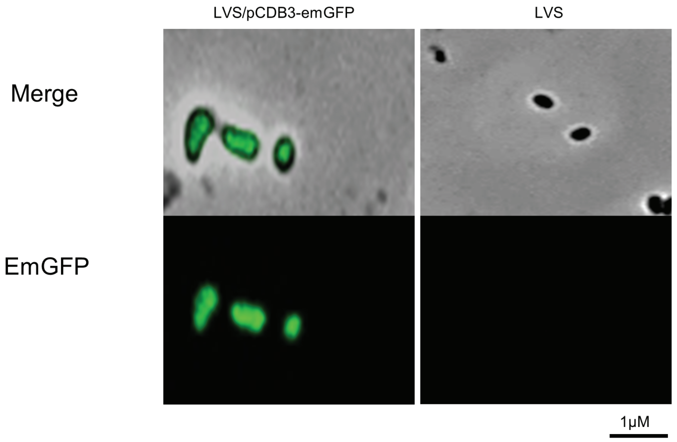

Figure S1. The recombinant cytoplasmic domain of Band 3 linked to emGFP is expressed throughout the cytoplasm of LVS. LVS/pCDB3-emGFP and LVS were grown in TSBc or Chamberlain's Defined Medium (CDM) to stationary phase at 37°C and added to pads containing 1% agarose in PBS. Expression of Band 3 linked to emGFP can be seen throughout the cytoplasm of LVS/pCDB3-emGFP cells.
