# Supplementary figures and images for: Host-pathogen interactions involved in erythrocyte invasion by Francisella tularensis
Source: Front Cell Infect Microbiol. 2025 Sep 23;15:1664733. doi: 10.3389/fcimb.2025.1664733 (PMC12500703; doi:10.3389/fcimb.2025.1664733)

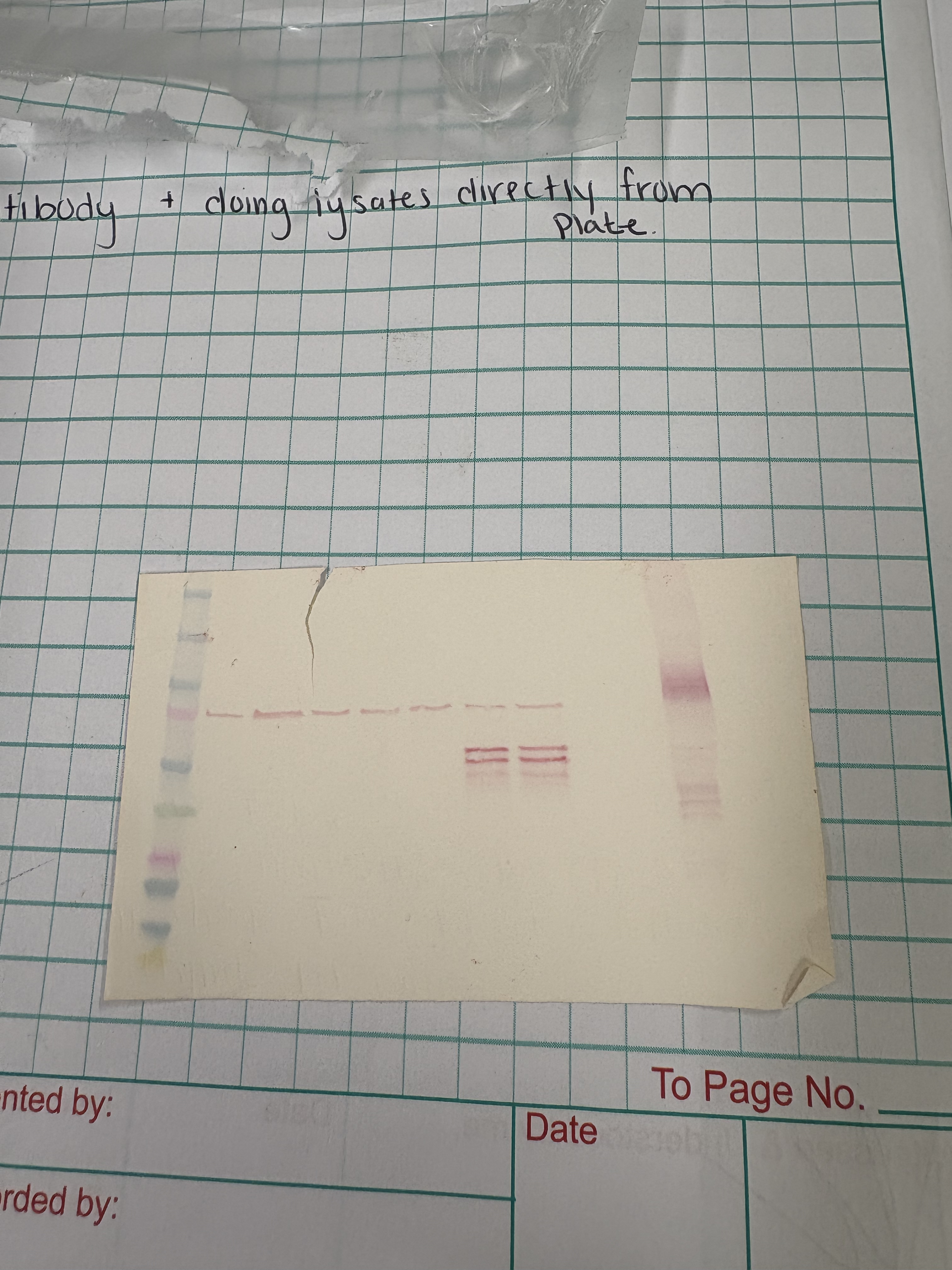

Supplement: Supplementary file 2 [file Image1.jpeg]

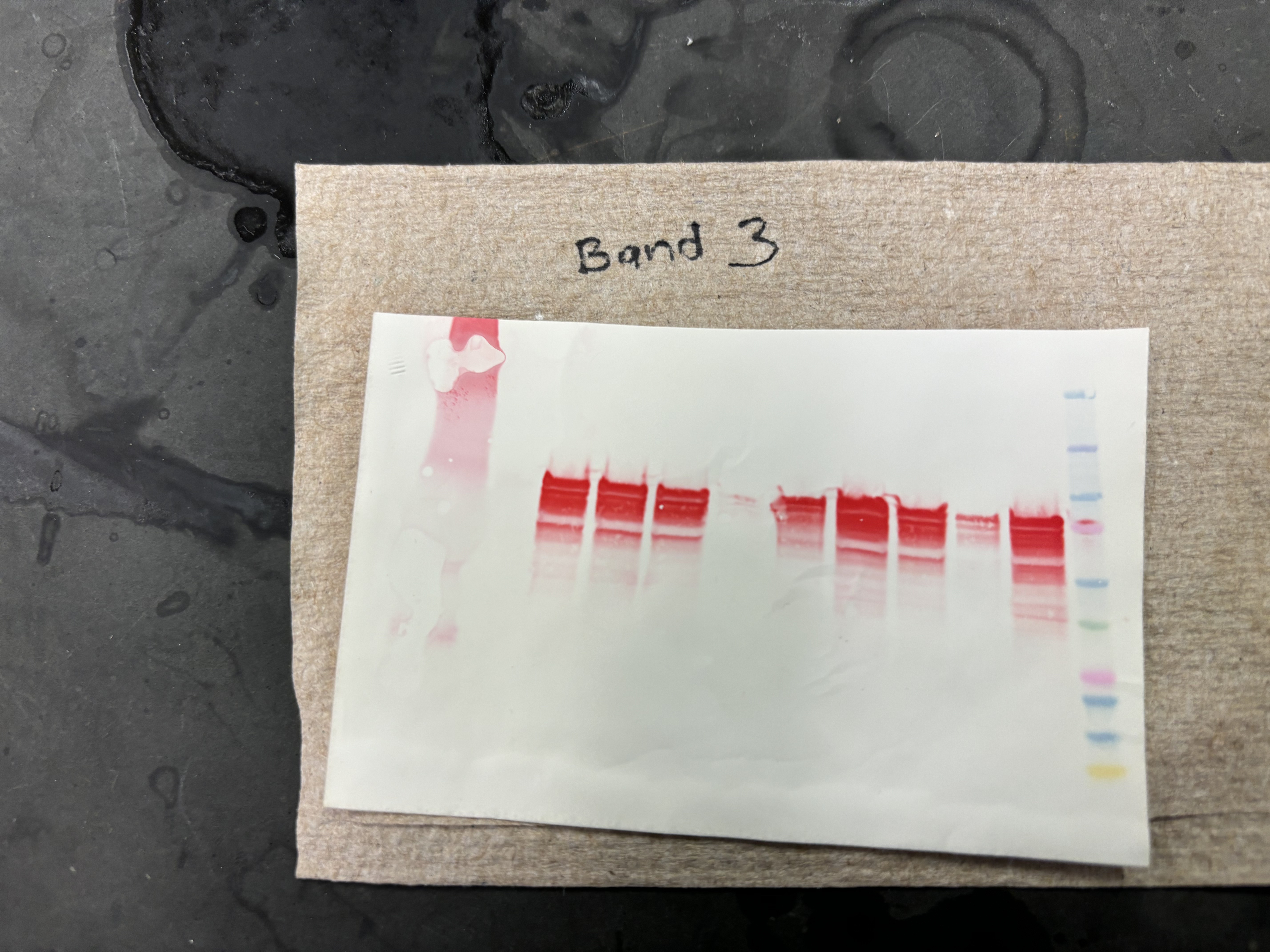

Supplement: Supplementary file 3 [file Image2.jpeg]

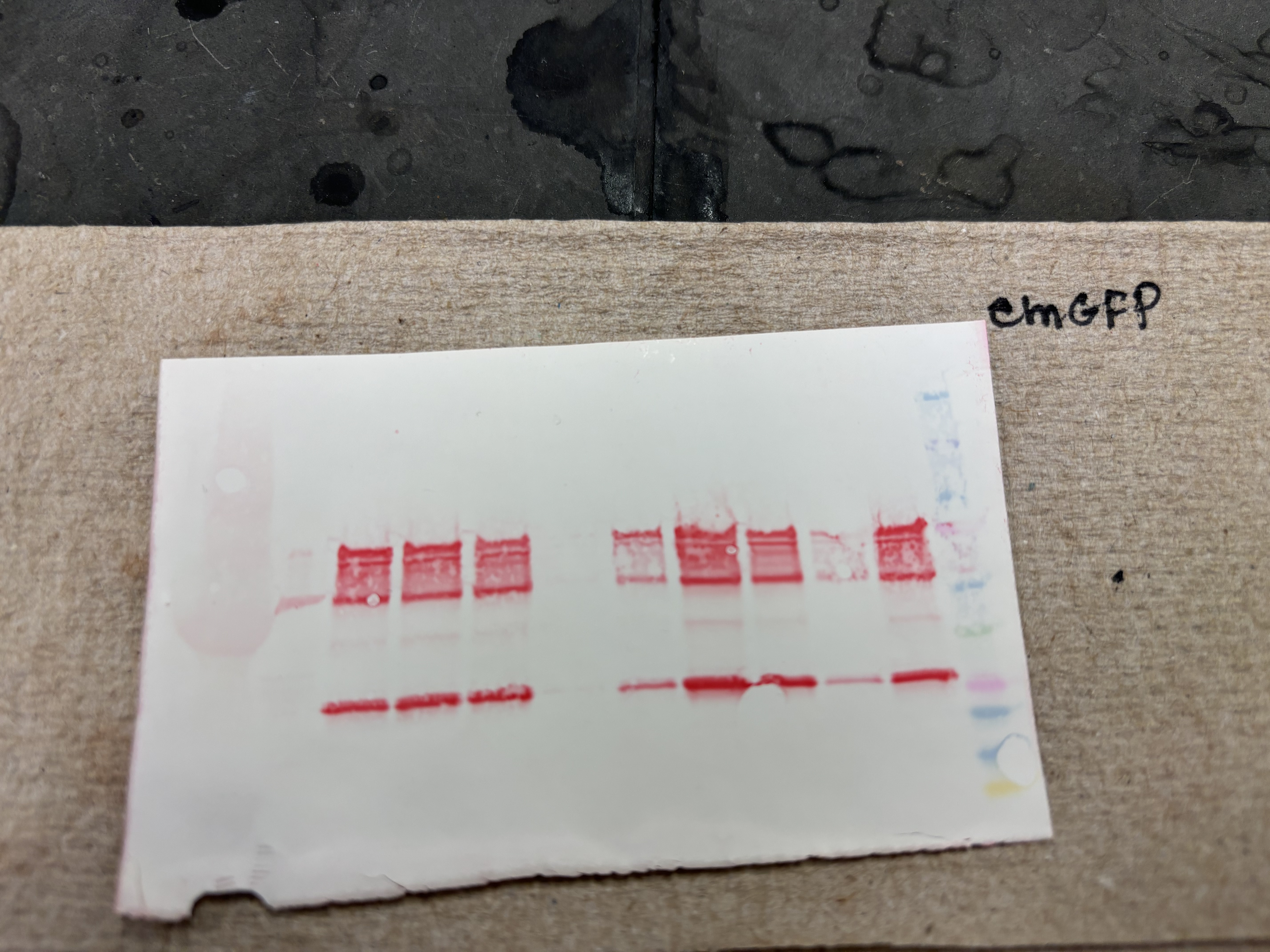

Supplement: Supplementary file 4 [file Image3.jpeg]

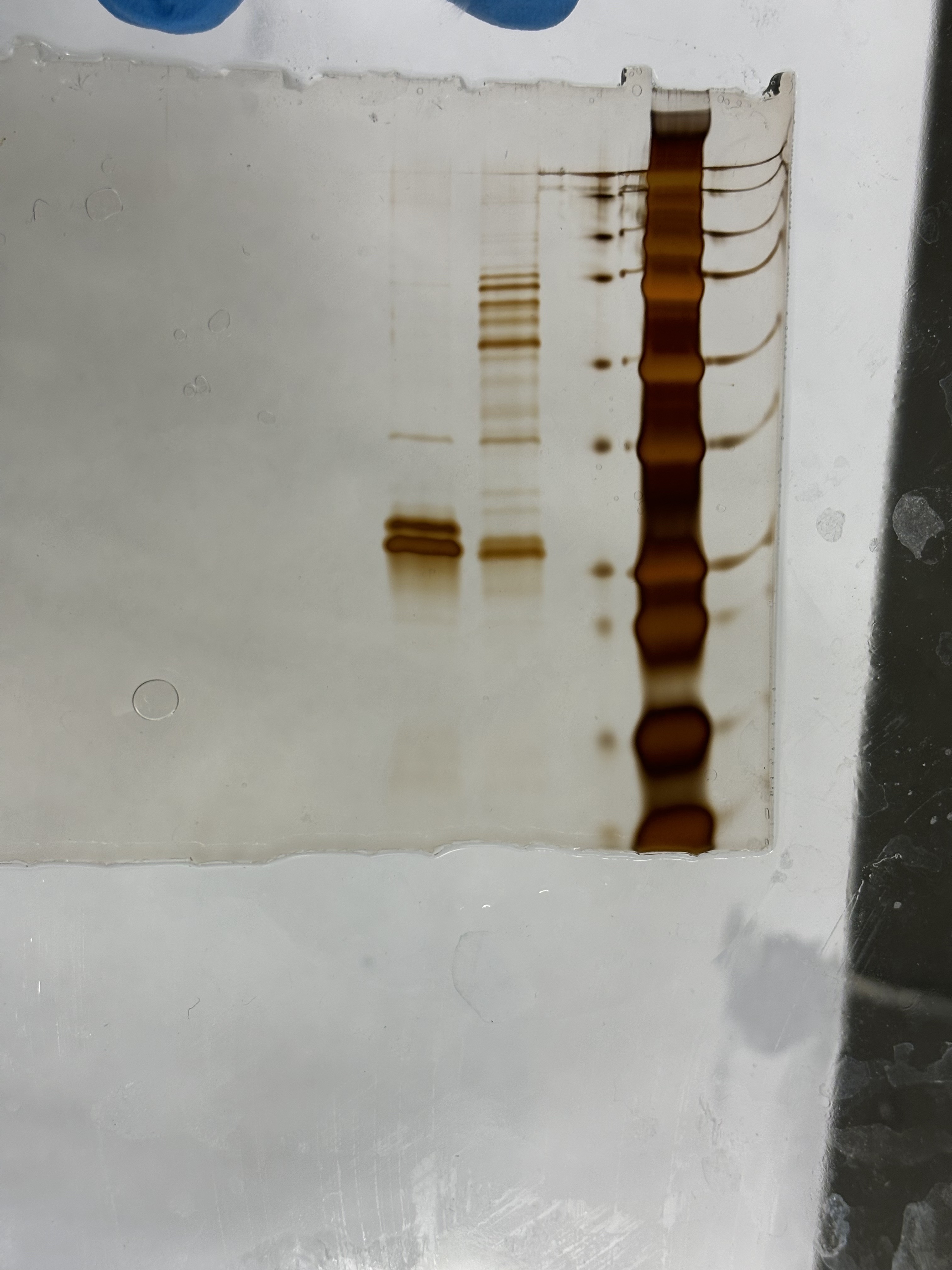

Supplement: Supplementary file 5 [file Image4.jpeg]

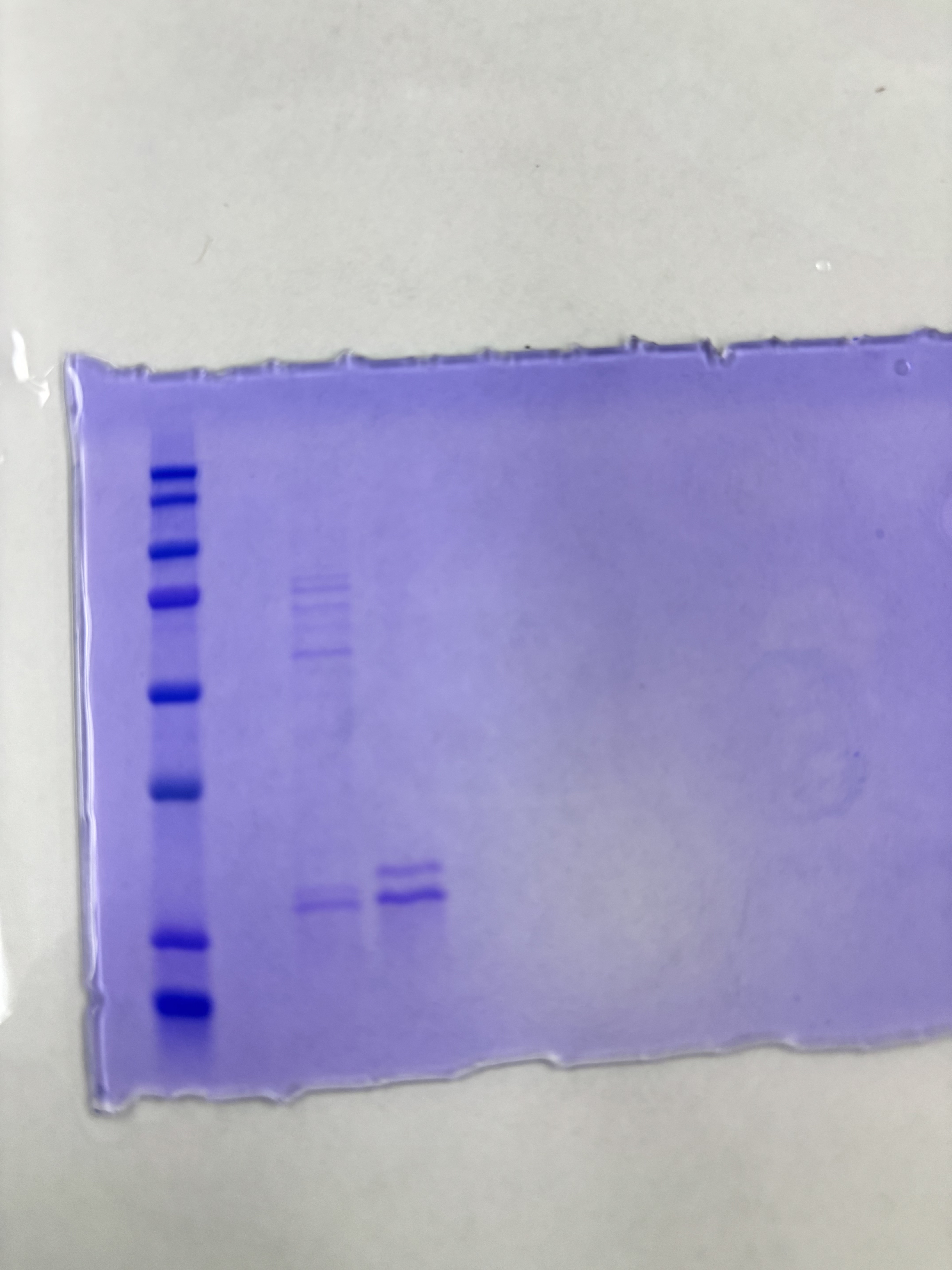

Supplement: Supplementary file 6 [file Image5.jpeg]

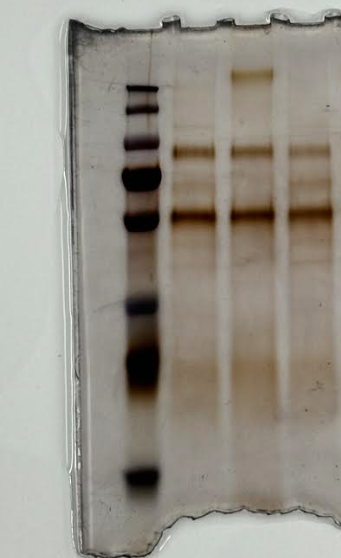

Supplement: Supplementary file 7 [file Image6.png]
